# Education and Cognitive Functioning Across the Life Span

**DOI:** 10.1177/1529100620920576

**Published:** 2020-08-10

**Authors:** Martin Lövdén, Laura Fratiglioni, M. Maria Glymour, Ulman Lindenberger, Elliot M. Tucker-Drob

**Affiliations:** 1 Aging Research Center, Department of Neurobiology, Care Sciences, and Society, Karolinska Institutet and Stockholm University, Stockholm, Sweden; 2 Department of Psychology, University of Gothenburg, Gothenburg, Sweden; 3 Stockholm Gerontology Research Center, Stockholm, Sweden; 4 Department of Epidemiology and Biostatistics, University of California, San Francisco; 5 Center for Lifespan Psychology, Max Planck Institute for Human Development, Berlin, Germany; 6 Max Planck UCL Centre for Computational Psychiatry and Ageing Research, Berlin, Germany, and London, United Kingdom; 7 Department of Psychology and Population Research Center, University of Texas at Austin

**Keywords:** educational attainment, cognitive ability, cognitive aging, life-span development, dementia

## Abstract

Cognitive abilities are important predictors of educational and occupational performance, socioeconomic attainment, health, and longevity. Declines in cognitive abilities are linked to impairments in older adults’ everyday functions, but people differ from one another in their rates of cognitive decline over the course of adulthood and old age. Hence, identifying factors that protect against compromised late-life cognition is of great societal interest. The number of years of formal education completed by individuals is positively correlated with their cognitive function throughout adulthood and predicts lower risk of dementia late in life. These observations have led to the propositions that prolonging education might (a) affect cognitive ability and (b) attenuate aging-associated declines in cognition. We evaluate these propositions by reviewing the literature on educational attainment and cognitive aging, including recent analyses of data harmonized across multiple longitudinal cohort studies and related meta-analyses. In line with the first proposition, the evidence indicates that educational attainment has positive effects on cognitive function. We also find evidence that cognitive abilities are associated with selection into longer durations of education and that there are common factors (e.g., parental socioeconomic resources) that affect both educational attainment and cognitive development. There is likely reciprocal interplay among these factors, and among cognitive abilities, during development. Education–cognitive ability associations are apparent across the entire adult life span and across the full range of education levels, including (to some degree) tertiary education. However, contrary to the second proposition, we find that associations between education and aging-associated cognitive declines are negligible and that a threshold model of dementia can account for the association between educational attainment and late-life dementia risk. We conclude that educational attainment exerts its influences on late-life cognitive function primarily by contributing to individual differences in cognitive skills that emerge in early adulthood but persist into older age. We also note that the widespread absence of educational influences on rates of cognitive decline puts constraints on theoretical notions of cognitive aging, such as the concepts of cognitive reserve and brain maintenance. Improving the conditions that shape development during the first decades of life carries great potential for improving cognitive ability in early adulthood and for reducing public-health burdens related to cognitive aging and dementia.

The duration of formal education and its importance for individual and social prosperity have rapidly expanded over the past 100 years (see Fig. 1). The growing importance of formal education has resulted from a combination of individual decisions, societal changes, and major policy initiatives. Although formal education is often considered most relevant to labor-market outcomes, educational attainment also has profound associations with individuals’ health throughout life. For example, as we review in this article, educational attainment is consistently related to both cognitive functioning and dementia risk in later adulthood.

**Fig. 1.**
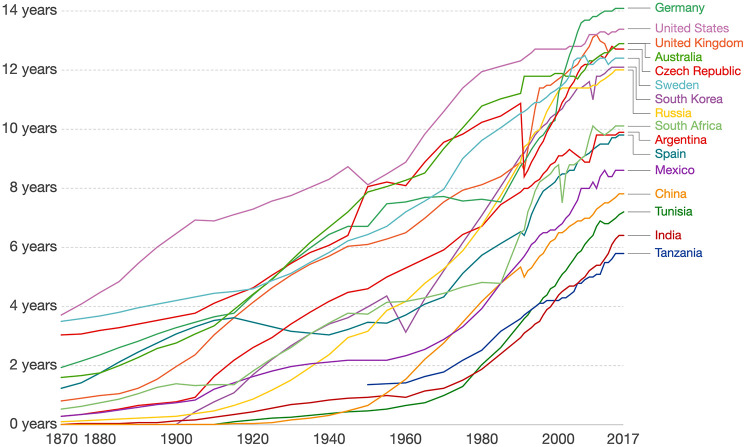
Average number of years of total schooling for individuals ages 25 years or older as a function of time (year), presented separately by country. From “Global Education,” by M. Roser and E. Ortiz-Ospina, 2020 (https://ourworldindata.org/global-rise-of-education), published under a CC BY 4.0 license.

In light of aging populations and increasing life expectancy, late-life cognitive impairment is a rising societal challenge, with estimates indicating that the global number of individuals with dementia will reach more than 130 million by 2050 (Prince et al., 2015). With effective disease-modifying treatments for late-life dementia currently unavailable, turning attention to potentially modifiable protective factors such as education is highly important. The implications of the association between educational attainment and late-life disease and functioning for both public-health policy and scientific theory, however, are dependent on the exact nature and causes of this association. Does longer education affect late life cognitive function and, if so, what are the mechanisms and moderators of that effect? Does educational attainment affect the rate of change in cognitive functions during late adulthood, or does it have effects on the development of cognitive functions in early adulthood that are then maintained into older age?

## Purpose

The purpose of this article is to review the literature on educational attainment’s associations with both levels of and changes in cognitive function in aging and dementia. This is not a systematic review but an attempt to provide an accessible synthesis of education’s role in cognitive functioning across adulthood. Recent analyses of data harmonized over multiple longitudinal cohort studies and related systematic reviews and meta-analyses form a solid ground for this synthesis.

We begin this article with a primer on the broad topic of cognitive functioning in aging and then go on to review the literature on observational associations between educational attainment and adult cognitive function. We caution readers against making strong mechanistic interpretations of the association at this stage of the review, providing detailed discussions of possible interpretations in later sections. As a variable, educational attainment is of course complex in many ways, and its associations with cognitive function may arise through a number of different causal processes. For example, (a) educational attainment may have a causal effect on cognitive development, such that increases in the duration of formal education cause increases in cognitive abilities; (b) educational attainment may be an outcome of preexisting cognitive ability, such that greater cognitive ability causes individuals to proceed further in education; or (c) external factors, such as family socioeconomic status, may affect both educational attainment and cognitive development. The initial portion of our review indicates that, irrespective of the relative importance of these different causal pathways, the association between educational attainment and cognitive function in adulthood is not specific to late life but is observed throughout adulthood, including early adulthood. We therefore focus the subsequent review on the mechanistic and developmental origins of the associations between education and cognitive ability. Finally, we synthesize the research to provide a general picture of the antecedents of this association and discuss its implications for our understanding of age-related changes in cognitive functioning and for public-health policy.

Notwithstanding the potential importance of nontraditional or informal educational experiences, our focus in this article is on formal, full-time education that is typically accumulated contiguously during childhood, adolescence, and early adulthood before long-term entry into the labor market. Separate reviews have been conducted on cognitive enrichment, cognitive training, and return to education in later life (e.g., Hertzog, Kramer, Wilson, & Lindenberger, 2009; Simons et al., 2016), and we refer to key issues and results in those areas only briefly in our synthesis of the findings toward the end of the article.

In research on aging, educational attainment is typically indexed by educational qualification or years of completed education. Educational qualification is measured on an ordinal scale, such as the International Standard Classification of Education, which sorts education into preprimary, primary, lower secondary, upper secondary, postsecondary nontertiary, first-stage tertiary, and second-stage tertiary levels. Years of completed education, typically after entry into primary education, is measured on an interval scale, but in some populations the variable is bounded at a lower level because of compulsory-education laws. Here, we use *educational attainment* to refer to both educational qualification and years of completed education. Defining educational attainment in this way elides many important distinctions, such as the quality of schooling, the social context of schooling, and the status associated with particular educational credentials. These differences may be just as important as the level or duration of education, yet research on such aspects to date is too sparse to allow for mature conclusions. The effects of years of schooling that we review here may therefore represent underestimates for highly enriched or intensive educational settings and overestimates for impoverished or less intensive settings.

## A Primer on Adult Cognitive Development

Cognitive abilities, measured both in childhood and in adulthood, are important predictors of life achievements, health, and mortality (Batty, Deary, & Gottfredson, 2007; Gottfredson & Deary, 2004; Schmidt & Hunter, 1998; Strenze, 2007). Within psychometrics and differential psychology, and particularly in the context of aging, researchers often distinguish between two broad classes of cognitive abilities (Baltes, Staudinger, & Lindenberger, 1999; K. B. Cattell, 1971; R. B. Cattell, 1987). Cognitive abilities that primarily rely on processing aspects of cognition are often referred to as *fluid abilities* (*Gf*) or cognitive mechanics. These abilities include psychomotor speed, memory, and abstract reasoning. (In some traditions, the term *Gf* is reserved for reasoning ability, but we use the term more broadly here to encompass abilities that influence reasoning performance, such as speed and memory.) Cognitive abilities that primarily reflect declarative and procedural knowledge explicitly acquired from one’s sociocultural environment are often referred to as *crystallized abilities* (*Gc*), or cognitive pragmatics, and include vocabulary, literacy, numeracy, knowledge of world history and current events, and specialized domain knowledge and skills.

Many tasks, both in the laboratory and in the real world, require a mixture of fluid and crystallized abilities. For instance, word-fluency tasks, in which individuals are asked to name as many words from a particular category as possible within an allotted amount of time, rely on both word knowledge and fluid abilities such as processing speed (Salthouse, 2005). Many complex professional and occupational tasks also likely require a mixture of specialized domain knowledge and fluid abilities, although individuals may modulate their reliance on different abilities to perform such tasks as they age and their balance of strengths and weaknesses changes (Baltes & Baltes, 1990; Salthouse, 1984).

All cognitive abilities, both fluid and crystallized, are moderately correlated with one another (Spearman, 1904). That is, differences between people are relatively consistent across different cognitive abilities, and people with high levels of one ability also tend to have high levels of another. Using factor analysis, this pattern of positive correlations can be summarized by a single common factor, known as *general intelligence*, or the *g* factor (Carroll, 1993; Spearman, 1904). General intelligence accounts for about half of the variation in individual cognitive-ability domains. Because of the correlations among various cognitive domains, it is common for researchers in aging to focus their attention on “general cognitive ability,” or simply “cognitive ability,” rather than many individual cognitive abilities. This focus on general cognition or a statistical summary of numerous domains is conceptually justified when researching determinants of cognitive function likely to have nonspecific effects—for example, factors such as nutrition that might have broad consequences for cognitive development across specific domains.

Although fluid and crystallized abilities are correlated across the life span, distinctions between them are particularly relevant in the context of aging (Baltes, 1987). Fluid cognitive abilities decline with advancing age during adulthood, even in the absence of detectable diseases. Longitudinal studies can measure within-persons development over time (i.e., change) by repeatedly assessing the performance of the same individuals. Change is then defined by, and computed from, the differences between the assessments (see Box 1). Such studies indicate that, on average, declines (i.e., there is a negative average rate of change; the second assessment of performance is lower than the first assessment) in performance begin in middle age or earlier and accelerate with age (Ghisletta et al., 2019; Rönnlund & Nilsson, 2006; Schaie, 1994, 2005). Crystallized abilities increase (i.e., they have a positive average rate of change; the Time 2 measurement of performance is higher than the Time 1 measurement) through middle age and are less adversely affected in older age (Rönnlund, Nyberg, Bäckman, & Nilsson, 2005; Schaie, 1994, 2005). Between-persons differences in cognitive abilities, however, are important in many settings, and those differences are becoming increasingly stable over the course of childhood (Bayley, 1949; Tucker-Drob & Briley, 2014) and are highly stable over extended periods (e.g., years) in middle adulthood (de Frias, Lövdén, Lindenberger, & Nilsson, 2007; Deary, Pattie, & Starr, 2013; Hertzog & Schaie, 1986, 1988; Tucker-Drob, Brandmaier, & Lindenberger, 2019; Tucker-Drob & Briley, 2014). In other words, between-persons differences in rates of change in cognitive abilities tend to be larger in early childhood and quite limited during adulthood until older age, at least in relation to between-persons differences in levels of cognitive abilities at any particular point in time. Nevertheless, small differences between people in the trajectories of cognitive changes during early and middle adulthood may be quite consequential, given that they may continue for several decades, and could lead to large differences in outcomes late in life.

**Box 1. “Change” During Development and Aging**

The term *change* has an important meaning in the study of development and aging. Change refers to within-persons development over time and can be measured only in longitudinal studies, which repeatedly assess a variable (two times or more), such as cognitive performance, in the same individual. Change is essentially defined as the difference between the measurements.In the case of two measurements, change equals the simple difference between the two measurements, typically computed by subtracting the scores from the first measurement (e.g., when a person is 20 years old) from the second measurement (e.g., when the same person is 70 years old). A positive value on the change variable thus represents an increase over time. A negative value on the change variable represents a decrease over time.A positive correlation between a variable—in this case, educational attainment—and cognitive change indicates that greater educational attainment is associated with a relatively more positive (i.e., less negative) cognitive change. For example, if longer education is associated with slower age-related decline of fluid cognitive abilities, then a positive correlation emerges. Table 1 gives an example of such a situation.A positive correlation between educational attainment and cognitive change can also indicate that greater educational attainment is associated with larger increases in cognitive performance over time (e.g., in a measure of crystallized intelligence such as vocabulary). Table 2 gives an example of such a situation.A negative correlation between educational attainment and cognitive change indicates that greater educational attainment is associated with less positive (i.e., more negative) cognitive change. For example, if longer education is associated with faster age-related decline of fluid cognitive abilities then a negative correlation emerges. Table 3 gives an example of such a situation.A negative correlation between educational attainment and cognitive change can also indicate that greater educational attainment is associated with smaller increases in cognitive performance over time (e.g., in a measure of crystallized intelligence such as vocabulary). Table 4 gives an example of such a situation.

**Table 1.** Example of a Positive Association Between Educational Attainment and Cognitive Change: More Education Is Associated with Smaller Age-Related Declines in Cognitive Performance

Years of education	Cognitive performance		Change (Time 2 – Time 1)	Correlation (education, change)
	Time 1	Time 2		
22	10	8	−2	.996
18	8	5	−3	
13	17	13	−4	
10	15	10	−5

**Table 2.** Example of a Positive Association Between Educational Attainment and Cognitive Change: More Education Is Associated with Larger Age-Related Increases in Cognitive Performance

Years of education	Cognitive performance		Change (Time 2 – Time 1)	Correlation (education, change)
	Time 1	Time 2		
22	10	15	5	.996
18	13	17	4	
13	5	8	3	
10	8	10	2

**Table 3.** Example of a Negative Association Between Educational Attainment and Cognitive Change: More Education Is Associated with Larger Age-Related Declines in Cognitive Performance

Years of education	Cognitive performance		Change (Time 2 – Time 1)	Correlation (education, change)
	Time 1	Time 2		
22	15	10	−5	−.996
18	17	13	−4	
13	8	5	−3	
10	10	8	−2

**Table 4.** Example of a Positive Association Between Educational Attainment and Cognitive Change: More Education Is Associated with Smaller Age-Related Increases in Cognitive Performance

Years of education	Cognitive performance		Change (Time 2 – Time 1)	Correlation (education, change)
	Time 1	Time 2		
22	8	10	2	−.996
18	5	8	3	
13	13	17	4	
10	10	15	5	

It is distinctly more difficult to predict between-persons differences in rates of cognitive change during aging than differences in levels of cognitive performance in older age. The reasons for that difference might be both methodological and substantive. Potential methodological reasons include lower statistical power to detect individual differences in change than to detect individual differences in levels of performance (Hertzog, Lindenberger, Ghisletta, & von Oertzen, 2006), which is likely to reflect a combination of smaller variances in change and a small number of longitudinal waves covering relatively short time spans (Brandmaier, von Oertzen, Ghisletta, Lindenberger, & Hertzog, 2018; Ghisletta et al., 2019). Potential substantive reasons include the possibility that individual differences in change arise from random processes or from processes that are themselves difficult to measure or are rarely measured—for example, human senescence may have an inherently random component (Kirkwood, 2005; Raz & Daugherty, 2018). Whatever the reasons, difficulties in predicting variation in cognitive change are pervasive. For example, although heritability of cognitive ability is high (e.g., Finkel, Pedersen, McGue, & McClearn, 1995), the estimates of heritability of changes in performance are more modest (e.g., Reynolds et al., 2005). Using commonly considered socioeconomic, genetic, lifestyle, and general health and fitness predictors in a linear model, Ritchie et al. (2016) reported that those predictors could account for only 16% of the differences between people in a general factor of longitudinal changes in cognitive abilities in older age, compared with 81% of variance in a general factor of baseline levels of cognitive abilities. In an earlier study, Albert et al. (1995) reported that a broad assortment of predictors explained a similarly low 25% of variance in cognitive changes. Note that even differences in cognitive function itself—whether they are indexed at the onset of a longitudinal study or during late childhood or early adulthood—have inconsistent and small, if any, associations with subsequent aging-related cognitive changes (Gow et al., 2012; Ritchie et al., 2016; Salthouse, 2012b; Tucker-Drob et al., 2019). That is, aging is not markedly kinder to the initially smarter.

In contrast to the weak associations between levels of cognitive function and subsequent changes in that function, different cognitive abilities have a strong tendency to change together over the course of aging. That is, longitudinal rates of cognitive change are correlated across different abilities (e.g., fluid and crystallized abilities; memory and speed). About two thirds of the variance in changes in different cognitive abilities is shared (Ghisletta, Rabbitt, Lunn, & Lindenberger, 2012; Tucker-Drob et al., 2019). Thus, just as there is a *g* factor of interindividual differences in different cognitive abilities measured at a single point in time, there is *g* factor of interindividual differences in changes in cognitive abilities over the course of aging. The consistent evidence that changes in highly disparate cognitive abilities are strongly correlated challenges the notion that individual differences in cognitive change are unreliable and weakens the proposition that longitudinal studies of cognitive aging are underpowered to detect correlates of change. This evidence is also scientifically valuable in its own right: It indicates that cognitive aging is a general phenomenon that pervades many different domains of cognitive function and suggests that a complete mechanistic account of cognitive aging cannot simply focus on domain-specific processes (Lindenberger, 2014; Salthouse, 1985). Rather, research into the mechanisms of cognitive aging will necessitate the identification of broad-ranging processes that have implications for many different cognitive abilities (e.g., Raz & Daugherty, 2018).

Changes in cognitive abilities during aging are associated with declines in the performance of everyday tasks that are important for independent living (Allaire & Marsiske, 2002; Tucker-Drob, 2011). Such reductions in performance might occur because cognitive declines constrain the range of tasks that individuals can successfully complete or constrain the range of abilities that individuals can use to complete tasks. Major associations between decreases in cognitive performance and changes in everyday functioning and well-being, however, are not always overt in independently living and professionally active older adults. That may be because older adults sometimes compensate by shifting to tasks that are less cognitively complex (e.g., they may avoid situations that reveal their deficits or challenge their maximum performance; Salthouse, 2012a) or by using abilities that remain relative preserved with age (e.g., acquired knowledge) to accomplish the same tasks and maintain a high level of performance (Baltes & Baltes, 1990; Salthouse, 1984).

Cognitive changes have particularly strong implications for everyday life when cognitive impairment is clinically diagnosed as dementia. According to the most widely used diagnostic criteria (American Psychiatric Association, 2013), dementia is a syndrome that requires decline in more than one cognitive domain with functional consequences for daily social or occupational activities. Clinically, dementia is most commonly classified as Alzheimer’s disease, which accounts for 50% to 70% of the cases, followed by vascular dementia, which accounts for 20% to 25% of the cases. Alzheimer’s disease is a neurodegenerative disorder characterized by insidious (i.e., gradual) onset and chronic progression due to an ongoing loss of neurons and synapses and consequent brain atrophy. Vascular dementia is diagnosed when dementia develops, often abruptly, after a stroke or in the presence of significant vascular brain alterations due to small-vessel disease. In contrast to this clinical classification, neuropathological and neuroimaging studies have shown that mixed pathologies often co-occur in the brain, and most individuals diagnosed with dementia after age 75 have multiple contributing pathologies (Savva, 2009; Schneider, Arvanitakis, Bang, & Bennett, 2007). Given that 70% of all dementia cases are diagnosed after age 75 (Winblad et al., 2016), it may therefore be pertinent to consider the role of their additive or synergistic interactions in producing the dementia syndrome than to view Alzheimer’s disease and vascular dementia (or other dementia subtypes) as dichotomous entities.

Because advanced age is the strongest risk factor for dementia, dementia and senescence are closely related (Drachman, 2007; Morris, Clark, & Vissel, 2018; Whalley, Dick, & McNeill, 2006). However, there is also strong scientific evidence that aging without dementia is possible, as shown by studies of secular (long-term) trends of dementia occurrence among centenarians (C. X. Qiu & Fratiglioni, 2018). Separating normative age-related cognitive changes from disease-related processes is often difficult, especially in the initial phase and at older ages, given the difficulties in assessing cognitive decline and functional independence in elderly people. Often, the diagnostic process is lengthy and requires several examinations during at least a 6- to 12-month period. Further, because dementia can be diagnosed only when clinical cognitive symptoms have become severe enough to cause functional declines in social and occupational activities beyond a lower threshold, it is essential to obtain information on individuals’ difficulty with daily activities from reliable informants. Because individuals of advanced age often have no relatives or other next of kin available, this can be problematic.

In recent years, several neuroimaging and biofluid indicators, or *biomarkers*, of neurodegenerative subtypes of dementia have been identified and even used in clinical practice (Winblad et al., 2016). Researchers in the field are currently debating the role that these biomarkers should play in the diagnosis of neurodegenerative disorders, including Alzheimer’s disease (Glymour et al., 2018; Jack et al., 2018). The major clinical limitation is the weak association between biomarkers considered hallmarks of neurodegenerative dementia disorders and cognitive measures relevant to everyday functioning in the early and prodromal phases of dementia’s progression. Major benefits of biomarker-based diagnostic systems are that they may help to identify more homogeneous clusters of dementia types for which treatment regimens can be more carefully tailored, and they may allow for early identification of pathology and treatment long before clinical manifestations of dementia are detectable.

**Fig. 2.**
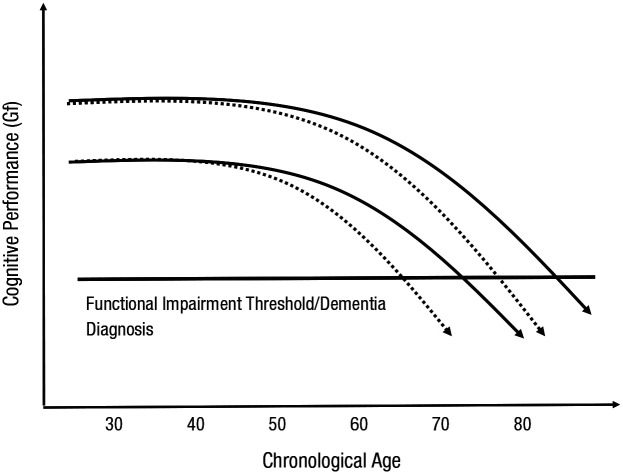
Schematic depiction of the importance of both levels of and changes in cognitive performance for understanding functional independence and dementia in older age. People reach functional-impairment thresholds at different points in older age because they start out with different levels of cognitive performance (compare the solid lines), because they experience different rates of cognitive change (i.e., different within-persons development; compare the solid lines with the dashed lines), or because of a combination of those differences. Initial differences between people in levels of performance can affect the age at which cognitive declines significantly interfere with daily life and, in turn, the point at which dementia is diagnosed (compare the two dashed lines). Gf = fluid abilities.

A full account of cognitive aging and dementia requires a focus on factors that shape (a) the levels of cognitive abilities attained by early adulthood and (b) the rates of cognitive change in adulthood and old age. To illustrate, imagine two individuals who have different cognitive-ability levels at age 20 but follow the same path through adult life thereafter. In line with the longitudinal evidence reviewed above, this path through adult life is characterized by a period of relative stability in early adulthood followed by period of accelerating decline as the individuals move into late adulthood and old age. These individuals will reach functional-impairment thresholds at different points in older age, although their rates of decline are identical (compare, for example, the solid lines in Fig. 2). Thus, developmental factors that give rise to peak levels of cognitive function in younger adulthood may affect the occurrence of cognitive impairment in later life, even when they have no effects on cognitive decline (compare the solid lines with the dashed lines in Fig. 2).

Even among individuals with particularly pronounced (clearly pathological) cognitive declines, initial differences in peak levels of performance may have a profound impact on the timing of significant interference in daily life and, consequently, diagnosis (compare the dashed lines in Fig. 2). For many individuals, cognitive declines that eventually give rise to a diagnosis of dementia may occur for years, even decades, before the diagnosis is made. All else being equal, this preclinical period will be longer for those who attained higher peak levels of cognitive function before the onset of cognitive declines. One implication of this extended preclinical phase of cognitive decline is that nearly any sample of older adults is likely to contain a proportion of individuals who are not yet diagnosed with dementia but will meet clinical diagnostic criteria within a few years, should they survive.

How do these issues relate to educational attainment’s association with late-life cognitive function and dementia risk? The level of a cognitive ability for a given person at any point in adulthood can be both heuristically and mathematically described in terms of that individual’s level of cognitive ability in early adulthood and his or her change from that level during the ensuing period (Hertzog, 1985). An association between educational attainment and cognitive function in older age may result from a relation between educational attainment and peak-level ability, age-related changes in ability, or some mixture of the two (see Box 1). Identifying an association between educational attainment and late-life cognitive function at a single point in time is not sufficient to distinguish among these possibilities. Moreover, dementia is diagnosed primarily on the basis of whether an individual’s general cognitive ability has declined below a threshold, such that daily social or occupational activities are affected. Therefore, associations between (a) education and level of performance or (b) education and change in performance are alone sufficient to account for an association between educational attainment and dementia risk (i.e., both can contribute to variation in when a threshold is reached; see Fig. 2). From a public-health perspective, both level of cognitive function and rate of decline in cognitive function influence an older individual’s quality of life, ability to live independently, and risk of mortality.

Of course, Alzheimer’s disease and other dementias are likely to have distinct etiologies (i.e., origins and causes) that partly separate them from other, more normative forms of cognitive aging. Researchers have therefore also attempted to describe cognitive changes surrounding dementia, and how they relate to educational attainment, in terms of two phases: cognitive changes leading up to diagnosis and declines in performance following a diagnosis. The rate of change, as measured with longitudinal cognitive assessments, reflects the combined effects of disease progression for all pathologies that are co-occurring in the brain, offset by repair or replenishment and compensation (Barulli & Stern, 2013; Cabeza et al., 2018; Nyberg, Lövdén, Riklund, Lindenberger, & Bäckman, 2012; Stern, 2002, 2006; Stern et al., 2018). These processes cannot be distinguished using conventional cognitive tests, but it is worth recognizing the distinctions because different intervention strategies may reduce the rate of disease progression or improve repair and compensation. In the next section, we assess the evidence for associations between educational attainment and both levels of and changes in cognitive functioning during aging.

## Evidence From Observational Studies

### Education and levels of cognitive function in the general adult population

Associations between measures of educational attainment and levels of cognitive function in adulthood are present in essentially all studied populations. These associations may result from a variety of causal mechanisms. In this section, we focus on the simple observational association between educational attainment and adult cognitive-ability levels. Later in this article, we return to questions of direction and modes of causation.

Two meta-analyses of the association between educational attainment and adult cognitive-ability levels are particularly relevant. Strenze (2007) conducted a meta-analysis of the association between intelligence (mainly measures of general cognitive ability from batteries of intelligence tests) in childhood through early adulthood (3–23 years) and education (highest degree attained or years of education) measured later in life (20–78 years). Fifty-nine samples (total *N* = 84,828) were included. The samples were restricted to Western populations, predominantly from the United States. The sample-size weighted-average correlation, corrected for error in measurement, was .56 (95% confidence interval [CI] = [0.53, 0.58]). The correlation increased with the age at cognitive assessment, from .42 when intelligence was tested between the ages of 3 and 10 years to .61 when it was tested between the ages of 19 and 23 years (confidence intervals for those correlations were not provided). Although the studies measured education over periods ranging from 1929 to 2003, no robust historical changes in these associations were evident (see also Hauser & Huang, 1997).

Focusing on older adults (age > 60 years), Opdebeeck, Martyr, and Clare (2015) conducted a meta-analysis of 109 studies (total *N* = 111,684) reporting estimates of the association between educational attainment and some measure of cognitive performance. The samples were mainly, but not exclusively, from Western populations. For general cognitive ability (typically composites across neuropsychological tests), the sample-size weighted-average correlation (not corrected for unreliability) was approximately .3. The effects were somewhat smaller for episodic memory and working or short-term memory (both correlations = .23) than for measures of (a) perceptual speed, (b) visuospatial ability, or (c) language abilities (all three correlations were around .3). Translating these effects into years of education indicates that 1 additional year of education is associated with cognitive performance that is roughly 0.04 to 0.08 *SD* higher. In other words, an individual with 5 additional years of education (e.g., a university degree) would be expected to have an advantage of about 0.2 to 0.4 *SD* in cognitive performance, or roughly 3 to 6 IQ points, relative to an otherwise comparable individual with less education (e.g., a high school diploma).

Several questions regarding the factors that may affect the magnitude of this association remain unanswered. For example, one might expect educational attainment to be more closely related to crystallized abilities, such as vocabulary and academic knowledge, than to fluid abilities, such as processing speed and abstract reasoning (e.g., Baltes et al., 1999; R. B. Cattell, 1987). In line with this notion, the association between measures of various aspects of language and educational attainment was among the strongest in the analyses by Opdebeeck et al. (2015). However, there were generally small differences, if any, between the cognitive domains. Differences in patterns of associations across different abilities may also be confounded by differences in the reliability of the ability measures. Without correcting for reliability differences, researchers cannot draw firm conclusions regarding different patterns of associations with educational attainment across cognitive-ability domains.

We also note that the meta-analyses discussed above were based on estimates of linear associations. However, nonlinear associations can occur for a variety of reasons. Finishing a degree may, for example, be associated with important outcomes beyond the continuous effects of time in school. Discontinuities associated with the receipt of credentials have been observed for some health outcomes, including mortality (Montez, Hummer, & Hayward, 2012). It is also possible that educational attainment and cognitive ability are nonlinearly related. For example, additional education may have diminishing marginal effects on cognitive ability at higher levels of ability or education. Moreover, given laws that prescribe minimum amounts of schooling and common socioeconomic barriers that curtail children’s education, variation in cognitive ability might play less of a role in educational attainment at lower education levels, in which mean educational attainment is likely to be closer to the mandated minimum.

Consider, for example, that compulsory schooling laws are strictly enforced in many countries, such that nearly all individuals attain a minimum basic level of education. Under a scenario in which educational attainment in the general population is partly determined by ability (i.e., more able individuals go further in school), we might expect a regression predicting educational attainment from cognitive-ability levels to underestimate the effect at the lower end of the education distribution because education is less an outcome of abilities at that lower range. By the same token, we might expect a regression predicting cognitive-ability levels from educational attainment to overestimate the effect at the lower end of the education distribution because some individuals with the minimum amount of schooling would have ability levels that would otherwise be associated with even less schooling (such that differences in cognitive performance between the minimum compulsory level and the next level of education would be larger than such differences between later levels of education). A few studies have reported such nonlinear effects—in particular, trends for a weaker association between education and cognitive performance after high school—but the magnitude of those effects is typically small, and it is noteworthy that the association remains positive at higher levels of educational attainment (e.g., Barnes et al., 2011; Myerson, Rank, Raines, & Schnitzler, 1998). The latter finding has also been reported for samples from non-Western societies (Kobayashi et al., 2017) and for health outcomes other than cognitive function (Montez et al., 2012).

Finally, we note that the association between educational attainment and cognitive function is robust across factors such as gender, race, society, and birth cohort (e.g., Kobayashi et al., 2017; Opdebeeck et al., 2015; Weber, Skirbekk, Freund, & Herlitz, 2014; Yang, Martikainen, Silventoinen, & Konttinen, 2016), although these factors may differentially influence the magnitude of the association—an issue we return to later in this review. Notwithstanding some uncertainty about the exact magnitude of the association between education and cognitive function, we can safely conclude that this association constitutes a highly consistent and replicable finding. Across the entire adult age range, individuals with more education show higher levels of cognitive function than individuals with less education.

### Education and aging-related cognitive changes in the general population of adults

The evidence reviewed above indicates that educational attainment is robustly associated with levels of cognitive function across adulthood. However, does educational attainment also relate to the rate of aging-related cognitive decline within the general population of adults? Two general approaches have been used to address this question. In the first approach, researchers use cross-sectional data (i.e., data for many subjects at one point in time) to examine whether the magnitude of associations between educational attainment and cognitive ability differs systematically with the age at which the abilities are assessed in adulthood (Hofer, Flaherty, & Hoffman, 2006; Hofer & Sliwinski, 2001). If educational attainment is associated with slower cognitive decline, we would expect the association between educational attainment and cognitive ability to strengthen as a function of age. To elaborate, with advancing adult age, variation in cognitive function should be increasingly determined by variation in aging-related cognitive changes. Therefore, if educational attainment is positively related to rates of cognitive aging (i.e., if greater education is associated with slower rates of cognitive decline; see Box 1), then the correlation between educational attainment and cognitive function should increase with age. In the second approach, researchers use longitudinal data to directly examine whether educational attainment is related to interindividual differences in rates of intraindividual cognitive change over time (i.e., if people with more education decline at a different rate than those with less education). We review evidence from studies employing each of these approaches.

The meta-analysis of young and middle-aged samples by Strenze (2007) produced a substantially larger estimate of the association between educational attainment and cognitive ability than the meta-analysis of older samples by Opdebeeck et al. (2015). The trend for a decreasing correlation with age, however, was also observed by Opdebeeck et al. (2015) in the older samples. That pattern is inconsistent with a protective effect of education on cognitive aging. Rather, it suggests that within-persons changes in cognitive ability are negatively related to education (i.e., that greater education is associated with faster cognitive declines; see Box 1). If education were protective, the association would increase with age, such that individual differences in performance would become more strongly related to education with age.

The observed cross-sectional pattern of decreasing correlations between education and cognitive ability with age is open to several alternative interpretations. For example, the neuropsychological tests dominating the data in the analyses by Opdebeeck et al. (2015), especially the measures of memory, typically have lower reliability (and may therefore indicate weaker associations with education) than the more well-established measures of general ability that were analyzed by Strenze (2007). Moreover, cohort differences, age-related changes in variance of cognitive-test scores, age differences in population representativeness, age-related differences in measurement reliability, and other methodological factors (as well as unknown differences between studies) are possible confounds in cross-sectional data. We therefore turn to directly evaluating the evidence for an association between education and within-persons longitudinal change.

The evidence for an association between educational attainment and longitudinal changes in cognitive function in aging is much more tenuous than that observed for associations between educational attainment and levels of performance. Several methodological issues may contribute to this mixed evidence. For example, appropriate statistical techniques for estimating change were not widespread until quite recently, and ceiling effects are severe in many cognitive measures commonly used in epidemiological studies (e.g., screening measures such as the Mini-Mental State Examination, or MMSE; Folstein, Folstein, & McHugh, 1975). Ceiling effects are particularly problematic when the predictor variable (education) is associated with differences in level of performance in the outcome variable. Indeed, some studies have reported negative correlations (e.g., Alley, Suthers, & Crimmins, 2007; Gross et al., 2015), others no correlation (e.g., Tucker-Drob, Johnson, & Jones, 2009; Zahodne et al., 2011), and yet others positive correlations (e.g., Arbuckle, Maag, Pushkar, & Chaikelson, 1998; Lyketsos, Chen, & Anthony, 1999) between educational attainment and cognitive change in older adults. (Note that a positive correlation between educational attainment and cognitive change indicates that higher educational attainment is associated with slower cognitive decline, whereas a negative association indicates that higher educational attainment is associated with faster cognitive decline; see Box 1.)

Reviews have also arrived at different conclusions. In a nonparametric meta-analysis of longitudinal studies of changes in cognitive performance that used screening instruments such as the MMSE, Valenzuela and Sachdev (2006) concluded that greater education is associated with slower declines in cognitive performance. In a narrative review of studies conducted through 1999, K. Anstey and Christensen (2000) arrived at the same conclusion, but a later narrative review of studies published since 1999 found a lack of consistent evidence that education is associated with age-related cognitive change (Lenehan, Summers, Saunders, Summers, & Vickers, 2014).

The development and more widespread application of appropriate statistical tools for analyzing longitudinal data (e.g., latent growth curve models in linear mixed or structural equation modeling frameworks) in recent years may partly explain why the bulk of more recent studies have arrived at different conclusions than earlier studies did. For example, many early studies adjusted for baseline cognitive performance using imperfectly reliable measures, and they often had only one repeated assessment (i.e., two assessment waves). Under those conditions, estimation of the effect of variables that are correlated with baseline cognitive performance (e.g., education) on change in cognition is biased, typically toward the cross-sectional association (Dugravot et al., 2009; Glymour, Weuve, Berkman, Kawachi, & Robins, 2005; Yanez, Kronmal, & Shemanski, 1998; see also Zahodne et al., 2011). Regression to the mean contributes to that bias. Informally, adjusting for baseline performance compares individuals with different educational levels but the same measured baseline performance. In that situation, there is an increased likelihood that at the baseline assessment, individuals with more education will show the same performance as individuals with less education or that individuals with less education will show the same performance as individuals with more education because of errors in measurement. At the follow-up assessment of cognition, the less educated individuals will be more likely to regress to the lower mean and the more educated individuals will be more likely to regress to the higher mean, which could falsely imply that the less educated individuals experienced greater declines.

Recent studies have circumvented that issue by estimating change with statistical methods (e.g., latent-growth-curve models, random-coefficient models, mixed-effects models, and multilevel models) that allow for estimates of change that account for influences of measurement error. Summaries of such studies have presented a more coherent picture of the association between education and late-life cognitive changes. In particular, recent efforts to harmonize data from different cohort studies have been valuable. A major study by Lipnicki et al. (2017) serves as a case in point. The authors harmonized and analyzed data from 14 longitudinal studies conducted in 12 countries (Australia, Brazil, France, Greece, Hong Kong, Italy, Japan, Singapore, Spain, South Korea, the United Kingdom, and the United States). Each study had 2 to 16 assessment waves (*M* = 3) and a follow-up duration of 2 to 15 years. A total of 42,170 individuals from 54 to 105 years old were included. The estimates pooled across the studies indicated that educational attainment had small associations with changes in language and processing speed and null associations with changes in memory and executive function (see Lipnicki et al., 2017, Table S16, which reports results excluding data for individuals with a dementia diagnosis at baseline). One extra year of education was associated with less decline in processing speed (0.008 *SD* per decade) but more decline on language measures (−0.008 *SD* per decade), and declines on measures of memory and executive functions were not statistically significantly associated with education (effect size = −0.003 *SD* for memory and 0.000 *SD* for executive functions). A very weak positive association (i.e., a protective effect) between education and change in memory was also reported in another major recent analysis of immediate- and delayed-memory scores for over 11,000 individuals from 10 countries who were 65 years or older and taking part in the Survey of Health, Ageing, and Retirement in Europe (Cadar et al., 2017).

**Fig. 3.**
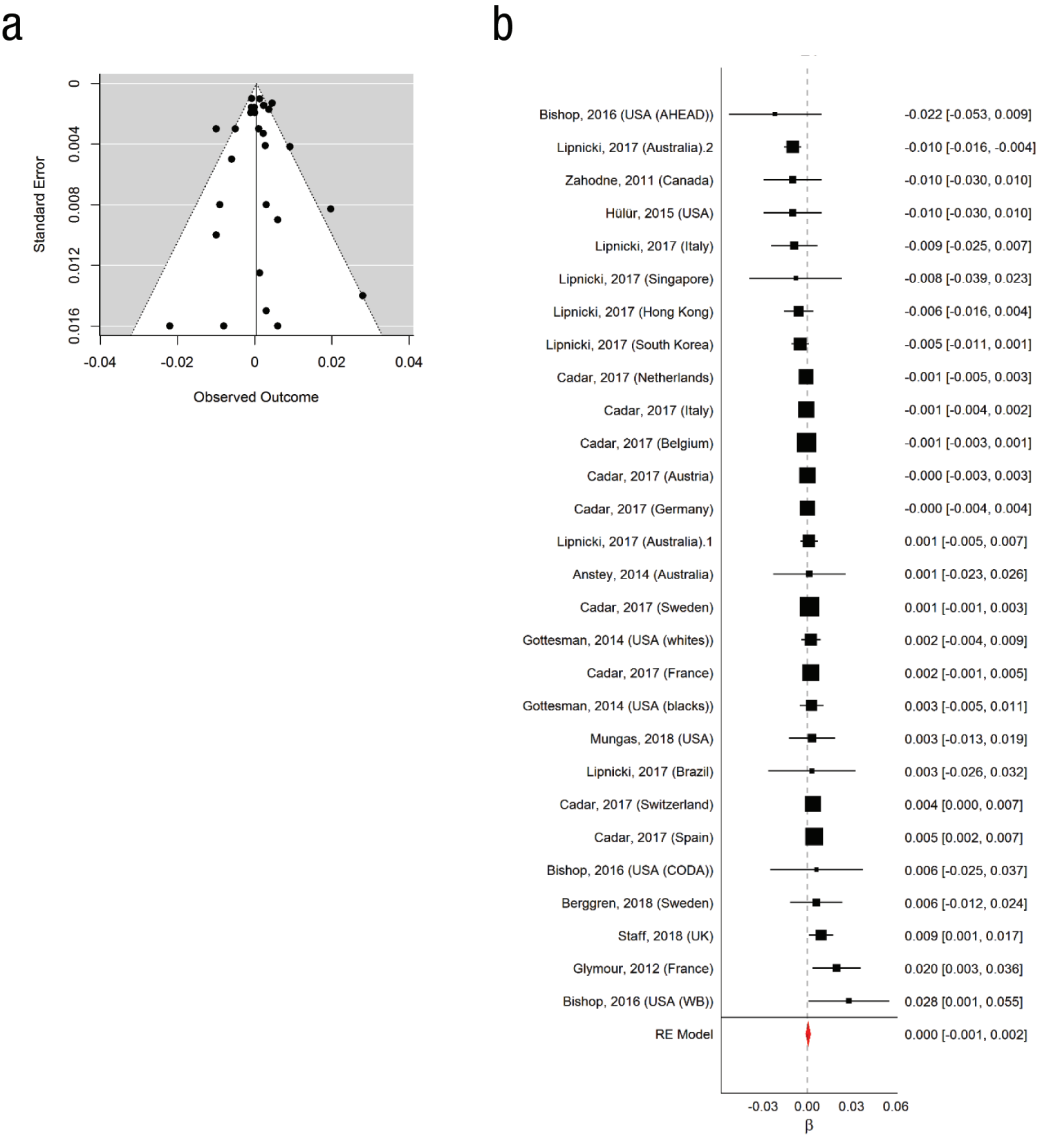
Funnel plot and forest plot of effect sizes (observed outcomes) from studies examining the association between education and age-related change in episodic memory. The funnel plot (a) shows standard errors as a function of effect size. Each plotted point represents a single study. The white triangle represents the region where 95% of the data points would lie in the absence of a publication bias, and the vertical line represents the mean effect size. The forest plot (b) shows correlations, represented by squares, reported for individual samples. The area of each square is proportional to the sample’s weight in the meta-analysis, and the horizontal lines represent confidence intervals (CIs). (The correlations and CIs are also provided in the right-hand column.) The red diamond shows the overall measure of the effect from a random-effects (RE) model. Results are shown with outliers removed; with outliers included, the average effect size was similar (−0.0003, 95% CI = [−0.004, 0.004]) to the one shown here (0.0005, 95% CI = [−0.001, 0.002]). Note that positive correlations between educational attainment and cognitive change indicate that greater educational attainment is associated with slower cognitive decline. A negative association indicates that greater education attainment is associated with faster cognitive decline (see Box 1). Adapted from “Education and Age-Related Decline in Cognitive Performance: Systematic Review and Meta-Analysis of Longitudinal Cohort Studies,” by D. Seblova, R. Berggren, and M. Lövdén, 2020, Ageing Research Reviews, Vol. 58. doi:10.1016/j.arr.2019.101005.

Against this background of small and inconsistent associations between education and longitudinal cognitive changes, as well as inconsistent results across measures, Seblova, Berggren, and Lövdén (2020) recently performed a meta-analysis to complement the streamlined, but less generalizable, analyses of harmonized data. Their results confirmed the general impression from the major recent studies. The point estimates of the average relationship between 1 additional year of education and cognitive changes were very small: less than 0.001 *SD* per decade for episodic memory (95% CI = [−0.001, 0.002]; Fig. 3) and for reasoning (or fluid ability; 95% CI = [−0.013, 0.013]), 0.002 *SD* per decade for general intelligence (95% CI = [−0.0003, 0.0051]), 0.002 *SD* per decade for processing speed (95% CI = [−0.002, 0.005]), −0.004 *SD* per decade for verbal fluency (95% CI = [−0.009, 0.0001]), and less than −0.001 *SD* per decade for crystallized ability (95% CI = [−0.004, 0.003]).^1^ None of these effects was statistically significant, despite the impressive precision of most of the estimates.

In summary, the association between education and cognitive change is in general likely to be small, even if small differences in rates of annual change may lead to substantive differences in levels of cognitive function over decades. We can use the meta-analytic point estimate for general intelligence to illustrate this. Over the two decades (from 60 to 80 years of age) during which cognitive performance shows the most marked decline (Rönnlund et al., 2005), an individual with a university degree (5 years of education beyond high school) is expected to experience approximately 0.02 *SD* (0.002 × 2 decades × 5 years) less overall decline than an individual with only a high school diploma. The effect estimated at the upper end of the 95% confidence interval raises that advantage to 0.05 *SD*. This is rather trivial compared with the average rate of episodic memory decline, which has been estimated around 0.4 to 0.5 *SD* per decade in this period of life (Berggren, Nilsson, & Lövdén, 2018; Rönnlund et al., 2005; Schaie, 1994). Note that the effect-size estimates for other cognitive abilities were all smaller than the estimate in this example. Overall, evidence indicates with high confidence that associations between education and rates of longitudinal cognitive decline are considerably smaller than the association between education and levels of cognitive function reviewed above (an advantage of 0.2–0.4 *SD* in cognitive performance, or 3–6 IQ points, associated with an additional 5 years of education—e.g., for an average individual with university degree compared with an average individual with only a high school diploma).

The meta-analysis reviewed above indicated substantial heterogeneity across studies in the magnitude of associations between educational attainment and changes in cognitive abilities, suggesting that a discussion of modifiers is warranted. Most previous studies have focused on older age, and it may, for example, be the case that an association is present during early adulthood. However, the reviewed meta-analysis (Seblova et al., 2020) and recent studies including an adult life-span sample (participants were 35–80 years old) have shown no strong indication that effects of education on cognitive ability are larger at younger ages (Berggren et al., 2018). It may also be that an association is nonlinear, such that effects of educational attainment on cognitive change may appear, for example, in samples of individuals who are poorly educated, but no such pattern was evident for any cognitive outcome examined in the reviewed meta-analysis. Some researchers have investigated whether education is associated with the trajectory of cognitive decline or the point at which it accelerates, and it is possible that such analyses may be more sensitive to effects of education than analyses that treat change as only linear.

Evidence for effects of education on points of accelerated change is mixed so far (Christensen et al., 2001; Clouston, Glymour, & Terrera, 2015; Clouston et al., 2019). The roles of factors such as turning points (e.g., retirement; Finkel, Andel, Gatz, & Pedersen, 2009), cohort effects (e.g., Karlsson, Thorvaldsson, Skoog, Gudmundsson, & Johansson, 2015), and retest effects that differ as a function of educational attainment (e.g., Christensen et al., 2001) are also not fully understood. Past studies also differed widely in their treatment of subjects with dementia. In some studies, such individuals were included in the analyses—in some cases, because the subjects were not screened for dementia at all. In other studies in which the researchers aimed to focus their analyses on normal-range (nonclinical) variation in cognitive aging, individuals with dementia diagnoses were excluded (though the quality of diagnoses varied widely from study to study). Even when studies excluded individuals with dementia using thorough diagnostic protocols, it is likely that individuals in the prodromal phases of pathological decline remained. It is important to note, however, that major studies in which the analyses both included and excluded dementia cases have not reported any major difference in the estimated associations between educational attainment and rate of cognitive change (e.g., Lipnicki et al., 2017). Finally, it is possible that associations between educational attainment and cognitive change differ across societies depending, for example, on the degree of equality in access to tertiary education, an issue we return to later in this review. No such evidence, however, emerged from the reviewed meta-analysis, which showed no association between effect sizes and the Gini coefficient (a commonly used measure of a country’s inequality). Thus, the sources of cross-study heterogeneity in the associations between educational attainment and age-related cognitive changes remain unknown.

Potential biasing effects of mortality and selective dropout also merit consideration (e.g., Foverskov et al., 2018; Glymour, Tzourio, & Dufouil, 2012; Johansson et al., 2004; Mayeda, Filshtein, Tripodis, Glymour, & Gross, 2018). Educational attainment has a robust association with survival, such that individuals with more education tend to live longer (Hamad, Elser, Tran, Rehkopf, & Goodman, 2018; Mackenbach, 2008). Mortality is a major cause of dropout in longitudinal studies, and it is possible that individuals who perish earlier experience faster cognitive decline in their final years than do comparably educated individuals who survive. Statistical analyses that are robust to violations of some assumptions regarding the random nature of selective dropout may, in some more extreme circumstances, fail to recover unbiased estimates of the association between educational attainment and longitudinal cognitive declines (Mayeda et al., 2018; Mayeda et al., 2016). However, studies that have attempted to address this issue have not reported substantially altered estimates (Foverskov et al., 2018; Glymour et al., 2012; Gottesman et al., 2014). We also note that in the meta-analysis described above, estimates of the association between educational attainment and cognitive change were related neither to participants’ age nor to the length of follow-up (Seblova et al., 2020). Given that age and length of follow-up would likely be associated with the extent of a bias introduced by selective dropout, these results do not suggest that selective dropout had a major influence on the estimates.

Despite several remaining research questions, the currently available evidence clearly indicates that the association between educational attainment and late-life cognitive changes is typically small, inconsistent, and practically less important than the association between educational attainment and levels of cognitive abilities.

### Education and dementia risk

**Fig. 4.**
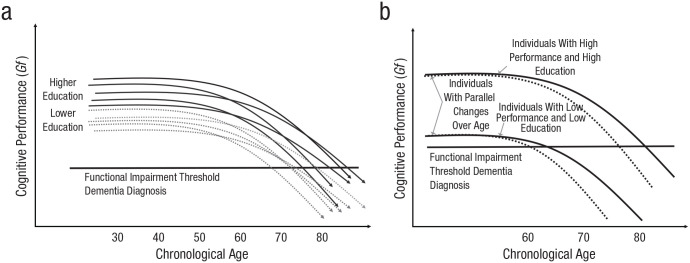
Schematics illustrating the relation between education and age-based cognitive decline. The graph in (a) summarizes evidence from observational studies indicating that individuals with higher and lower levels of education tend to differ in cognitive ability in early adulthood and, on average, show only small differences in rates of cognitive decline over time. As a result, more highly educated individuals pass a threshold for functional impairments and dementia diagnoses at a later age. Note that if the sample for a study is selected on the basis of a threshold of low performance at a particular age (e.g., 80 years old), then highly educated individuals experiencing a nonrepresentative sharp decline in performance will be overrepresented. The graph in (b) is a more extreme schematic, for illustrative purposes, of the association between education and cognitive performance in adulthood. Note that highly educated and less educated people (with corresponding performance) differ in their rate of cognitive change as they approach the threshold for a dementia diagnosis (more highly educated individuals show faster declines at a later age), although they show parallel cognitive trajectories. This is also the case for individuals with more marked (and pathological) decline than in the general population—for example, individuals who will be diagnosed with Alzheimer’s disease (compare the dashed lines in b). The origin of this effect is the acceleration of decline. Gf = fluid abilities.

By itself, an association between level of cognitive function and educational attainment indicates that dementia incidence is related to educational attainment. Even all else being equal, differences in peak levels of cognitive function during early adulthood are expected to lead to differences in when cognitive function declines below a threshold beyond which daily functioning is substantially impaired. For example, consider two individuals who differ in their levels of educational attainment, and hence their peak levels of premorbid cognitive function, and who progress along parallel trajectories of accelerating cognitive decline. Should they survive long enough, each will eventually reach a lower level of functioning beyond which a dementia diagnosis becomes probable. Because the more educated individual started his or her trajectory of decline from a higher peak level of cognitive function, he or she will reach the functional threshold at a later age (see Fig. 4). Indeed, since the early 1990s, several population-based studies have reported an increased risk for dementia among adults over the age of 65 with low educational attainment (Sharp & Gatz, 2011). Systematic reviews and meta-analyses have confirmed that association (Beydoun et al., 2014; Caamano-Isorna, Corral, Montes-Martinez, & Takkouche, 2006; Fratiglioni & Wang, 2007; Meng & D’Arcy, 2012; Valenzuela & Sachdev, 2006; X. J. Wang et al., 2019; Xu et al., 2015; Xu et al., 2016).

In their extensive review, Meng and D’Arcy (2012) were able to identify and analyze 50 population-based studies exploring this topic using prevalent (existing) dementia cases and 22 reports involving incident (new, or newly diagnosed) dementia cases. In two separate pooled analyses of the prevalence and incidence studies, respectively, they found a 2.6-fold (95% CI = [2.2, 3.1]) and 1.9-fold (95% CI = [1.5, 2.3]) increased risk for dementia in less educated compared with more highly educated people. Further, 18 of 20 additional prevalence studies reported a similar association between low educational attainment and dementia risk, although those studies could not be used in the pooled analysis because of a lack of suitable statistical estimates. Finally, a more recent meta-analysis of 15 longitudinal studies investigated the dose-response association between educational attainment and dementia risk (Xu et al., 2016). The overall trend suggested that education reduces dementia risk in a relatively linear dose-response manner. However, few of the individual studies that were included in the meta-analysis showed a clear dose-response pattern through the whole range of years of schooling. In addition, data on the association between tertiary versus secondary education and dementia risk are relatively scarce. Thus, further work is needed on whether education is associated with dementia risk across all durations or levels of education. In sum, low educational attainment is associated with greater incidence of dementia at any age and, consequently, with an earlier age of dementia diagnosis. This is consistent with an association between educational attainment and peak levels of cognitive function in early adulthood.

### Education and cognitive decline before and after dementia diagnosis

Several studies have investigated whether education modifies the progression of cognitive changes leading up to and following a dementia diagnosis. These studies have either retrospectively or prospectively charted trajectories of cognitive change surrounding dementia diagnoses. As we discuss below, these studies need to be carefully interpreted because they violate the basic analytic dictate not to restrict analyses on the basis of factors (dementia) that are consequences of the dependent variable (cognitive performance). Such studies also face the more prosaic risk of education-related ascertainment and diagnostic bias (e.g., differential care-seeking behavior and access to health care may cause some people to be screened or diagnosed at disproportionate rates); for that reason, they should be structured as longitudinal cohort studies, employ careful diagnoses of dementia, and base their data on incident cases. Such studies also naturally have dense longitudinal data on cognitive performance before dementia diagnosis.

A systematic search for studies investigating whether education is related to the progression of cognitive changes before a diagnosis of dementia revealed only seven nonoverlapping reports (total *N* = 3,323).^2^ The type of analysis, measure of cognitive function, and availability of effect sizes varied widely across studies. Five of the studies (total *N* = 2,156; average *N* = 431; range = 117–856) reported faster decline for more highly educated individuals than for less educated individuals on a measure of cognitive function during the years immediately before dementia diagnosis (approximately 3–5 years). For instance, in the Personnes Agées Quid (PAQUID) cohort, individuals who had completed primary school, compared with those who had not, showed more rapid declines on measures of verbal fluency, psychomotor speed, and, in particular, episodic memory (Amieva et al., 2005; Amieva et al., 2014; Jacqmin-Gadda, Commenges, & Dartigues, 2006). A few studies have reported a later acceleration but faster decline after a modeled change point for more highly educated individuals on measures of episodic memory (Hall et al., 2007) and general cognitive ability (but see also C. Li, Dowling, & Chappell, 2015; Yu et al., 2012). Scarmeas, Albert, Manly, and Stern (2006) reported faster declines among more highly educated subjects before dementia diagnosis on measures of executive functions, psychomotor speed, and general cognition (but not measures of language or visuospatial ability). In the large Rotterdam Study (*N* = 856), performance on the MMSE declined faster among more highly educated individuals than among less educated individuals in the years before they were diagnosed with dementia (Verlinden et al., 2016). Two studies detected no statistically significant effect of education on cognitive change in the years before dementia diagnosis. In the smaller study (*N* = 127), no effects were detected on a wide range of cognitive measures (Cadar, Piccinin, Hofer, Johansson, & Muniz-Terrera, 2016). In the larger study (*N* = 1,040), which was the largest conducted so far, education modified neither the point of accelerated decline before a dementia diagnosis nor the speed of the decline on a measure of general cognition (G. Li et al., 2017).

Overall, then, studies examining associations between educational attainment and cognitive declines surrounding dementia have been limited, and their results have been mixed. We can nonetheless discern a trend toward faster decline for more highly educated individuals in the years immediately before a dementia diagnosis; there is some strong conflicting evidence of null effects, but no evidence for the opposite effect. It is likely, however, that this trend is fully explained by two statistical artifacts stemming from how educational attainment relates to peak levels of cognitive abilities in early adulthood, before dementia. Those artifacts are *collider bias* and *ascertainment timing*. To understand both, consider first the premises that are clear from our review so far: (a) Individuals with different levels of education should, on average, differ in (premorbid) peak ability levels in early adulthood, before experiencing declines in cognitive functions that accelerate toward the threshold for a dementia diagnosis; (b) at the overall population level, individuals with different amounts of education should show only minor differences in their rate of cognitive decline over time; and (c) because dementia is primarily diagnosed on the basis of a decline below a functional threshold, individuals with more education should, all else being equal, receive a dementia diagnosis at later ages, on average. This is the scenario depicted in Figure 4a and, in a more extreme way (for illustrative purposes), in Figure 4b.

The term *collider bias* is generally used to refer to situations in which two variables that jointly determine an outcome become artifactually correlated (often inversely correlated) in analyses selecting on or controlling for that outcome. For example, conscientiousness (i.e., work ethic) and IQ (aptitude) each contribute to admission to selective colleges, such that the two variables are negatively correlated in samples of college students even though they are slightly positively correlated in unselected samples (Murray, Johnson, McGue, & Iacono, 2014). In the current context, peak (premorbid) ability levels and the rate of decline since that peak jointly determine the age at which an individual’s cognitive functioning will decline beyond the threshold for a dementia diagnosis. Because studies of cognitive change surrounding the onset of dementia necessarily include only individuals who have been diagnosed with dementia, a collider bias with respect to peak level of cognitive functioning (and its determinants, such as educational attainment) and rate of cognitive decline is introduced.

To understand how that bias arises, consider how a researcher would select individuals for a dementia-based analysis from those whose trajectories are depicted in Figure 4a. Individuals would be included only if their cognitive performance declined below the threshold for a diagnosis by a particular age (e.g., 80 years) or time in the study. Thus, most of the individuals in the low-educational-attainment group would be included in the analysis, but individuals in the high-educational-attainment group would be included only if they had experienced particularly pronounced cognitive declines. That indirect selection on the basis of education would result in an association between educational attainment and rates of cognitive decline even if no such association exists in the population at large. Highly educated individuals who do not experience fast cognitive declines are the least likely to have crossed the threshold beyond which a dementia diagnosis is probable and would therefore be most likely to be missing from the sample. It follows that the link between greater educational attainment and faster cognitive decline before dementia diagnosis is induced, as a methodological artifact, by restricting the analysis to individuals who have been diagnosed with dementia. Indeed, empirical results have indicated that, in dementia studies, the more educated groups are less representative of the population than are the less educated groups (Amieva et al., 2014).

We use the term *ascertainment timing* here to refer to when in the process of cognitive decline dementia is diagnosed. Simply as a result of having commenced their cognitive declines from a higher peak level of premorbid cognitive function, more highly educated individuals are expected to be older (and hence in later stages of decline) than less educated individuals when they receive a diagnosis, even if the onset and the rate of cognitive decline are held constant (see Fig. 4a and, for an extreme depiction, Fig. 4b). If cognitive declines accelerate as a function of age, even if that acceleration is unrelated to educational attainment, then the later average age of dementia diagnosis among more highly educated individuals will necessarily lead to ascertainment during a period of faster cognitive decline (see Fig. 4b). Thus, the trend of sharper decline before a dementia diagnosis among more highly educated individuals in some studies could very well have arisen simply as a function of the relation between educational attainment and peak cognitive-ability levels during early adulthood.

Figure 4b illustrates how such differences in rates of change by educational-attainment levels would be expected to continue after dementia was diagnosed. More highly educated individuals are expected to be at a later, and therefore more rapid, phase of cognitive decline following their dementia diagnosis simply by virtue of having begun their trajectories of decline from a higher level of initial ability. A systematic search for studies investigating whether education is related to the progression of cognitive changes after dementia diagnosis revealed eight nonoverlapping studies (total *N* = 1,815), four of which (total *N* = 555) indeed reported faster declines for more highly educated individuals on measures of cognitive function during the years immediately after a dementia diagnosis). Of those studies, two reported data from very small samples (Contador, Bermejo-Pareja, Pablos, Villarejo, & Benito-Leon, 2017; Unverzagt, Hui, Farlow, Hall, & Hendrie, 1998). However, Andel, Vigen, Mack, Clark, and Gatz (2006) studied a larger sample of 171 individuals with a diagnosis of Alzheimer’s disease in a longitudinal cohort study. Individuals with a high level of education (> 12 years) showed faster declines on the MMSE, controlling for age and measured dementia severity at baseline, than did those with a lower level of education. In the largest study (*N* = 312), Scarmeas et al. (2006) reported faster cognitive decline among more highly educated subjects after a dementia diagnosis on measures of executive functions, speed, and general cognition (see also Stern, Albert, Tang, & Tsai, 1999, for data from a subpopulation). Three studies (total *N* = 587) reported no education-related differences in declines in performance on the MMSE (Aguero-Torres, Fratiglioni, Guo, Viitanen, & Winblad, 1998; Small, Viitanen, Winblad, & Bäckman, 1997; Tschanz et al., 2011).

A study of 670 individuals with a diagnosis of Alzheimer’s disease by Bruandet et al. (2008) is a special case in point. In univariate analyses, education was not related to declines in performance on the MMSE. When the researchers controlled for a large number of factors, a difference in the acceleration of change emerged, but it was largely driven by a single data point. It is therefore hard to draw any conclusions from this study. Of course, as described above, studies that rely on dementia screening instruments, such as the MMSE, suffer from additional biases associated with ceiling effects. A recent study by Jutkowitz et al. (2017) is also worth mentioning, although the sample (*N* = 457) was composed of participants from a prospective cohort study and self-referrals. In linear mixed analyses controlling for age, educational attainment was associated with higher MMSE scores at the time of diagnosis, but there were no education-related differences in decline 8 years thereafter. In the context of studies of patients after diagnosis, a major recent study of 4,500 individuals in placebo groups of randomized controlled drug trials deserves attention. Individuals with more education showed faster 12-month declines in performance on the Alzheimer’s Disease Assessment Scale–Cognitive subscale, an extensive measure of general cognition (Thomas, Albert, Petersen, & Aisen, 2016). However, possible education-related ascertainment bias and a lack of data on the baseline differences in cognitive function between people with more education and less education limit the interpretation of those results.

In sum, data on the progression of cognitive impairments before and after a dementia diagnosis are scarce and difficult to interpret. Some studies indicate faster deterioration of cognitive functions among more highly educated individuals. That pattern of results can be accounted for by methodological biases introduced by study designs and by the population-level pattern of education-related differences in peak cognitive-ability levels during young adulthood, as depicted in Figure 4.

### Summary of the evidence from observational studies in the broader context of cognitive aging and dementia

To summarize the empirical evidence from observational studies: Although some uncertainties remain, a broad picture of how education relates to cognitive aging is emerging quite clearly (Fig. 4a). Throughout adulthood, cognitive function in individuals with more years of schooling is, on average, better than cognitive function in those with fewer years of schooling. This association between educational attainment and cognitive function is robust and substantial and persists into late adulthood. The present evidence also indicates that this association holds across a wide range of educational levels. At the same time, the available evidence indicates that education has small and inconsistent associations with cognitive changes throughout adulthood and old age. Taken together, the two findings warrant the conclusion that the link between education and adult cognitive-ability levels is practically much more important than the link between education and aging-related cognitive changes. To be clear, despite the lack of a consistent and appreciable association between educational attainment and aging-related cognitive change, education has important implications for cognitive aging. The education-level associations are maintained into older age, thus serving as substantial source of differences among older people. These differences influence the ages at which thresholds for cognitive impairments are reached. As a result, education is a robust predictor of age of dementia onset that has a sizable effect.

The picture painted by our review so far is broadly consistent with other empirical findings in the field of cognitive aging. For example, between-persons differences in cognitive performance are remarkably stable throughout adult life (de Frias et al., 2007; Deary et al., 2013; Hertzog & Schaie, 1986, 1988; Tucker-Drob & Briley, 2014; Tucker-Drob et al., 2019). More sizable individual differences in change tend to emerge in older age (de Frias et al., 2007; Tucker-Drob & Briley, 2014), but, as reviewed above, these differences are difficult to predict. For example, level of cognitive performance has small and inconsistent associations with subsequent cognitive change (Gow et al., 2012; Salthouse, 2012b; Tucker-Drob et al., 2019). This finding is in line with the lack of a substantial association between education and cognitive change.

The substantial association between education and level of cognitive function, but not change in cognitive function, is also consistent with the observation that the massive increases in cognitive performance across birth cohorts (the so-called Flynn effect; Flynn, 1984; Rönnlund & Nilsson, 2008; Schaie, 1965, 2005) have not been accompanied by consistent birth-cohort effects in rates of cognitive aging. Intergenerational increases in cognitive function may partly be attributed to secular increases in educational attainment over the past century. However, there is little evidence that these birth-cohort effects translate into clear intergenerational differences in rates of change in performance: Later-born cohorts do not appear to generally show slower aging-related cognitive decline. In fact, although several methodological issues remain to be dealt with (e.g., accounting for changes in dementia incidence) and the evidence base is currently small, some studies have actually shown faster declines in later-born cohorts (Hulur, Infurna, Ram, & Gerstorf, 2013; Karlsson et al., 2015). Other studies, however, have shown slower declines (Gerstorf, Ram, Hoppmann, Willis, & Schaie, 2011) or no differences (Finkel, Reynolds, McArdle, & Pedersen, 2007). Moreover, life-span cognitive data from societies with very low overall levels of education show the same qualitative patterns of changes observed in societies with higher average educational attainment (Gurven et al., 2017).

A few prospective genetic, lifestyle, and general health and fitness predictors of age-related cognitive change have been identified. These include later-life physical and social activity (e.g., Blondell, Hammersley-Mather, & Veerman, 2014; Lövdén, Ghisletta, & Lindenberger, 2005), physical fitness (e.g., walking speed, grip strength; e.g., Ritchie et al., 2016), and cardiovascular risk factors (e.g., R. Wang et al., 2015). Many of these factors are tightly linked to physical health (and chronological age). There are fewer findings of robust and sizable associations between factors closely linked with educational attainment (e.g., socioeconomic conditions) and longitudinal cognitive declines, although some evidence suggests later-life socioeconomic factors may relate to the rate of memory decline (Marden, Tchetgen Tchetgen, Kawachi, & Glymour, 2017). The joint predictive power of those factors that are related to the rate of cognitive change is modest, especially in comparison with their much greater success in accounting for variation in levels of cognitive performance (Albert et al., 1995; Ritchie et al., 2016).

Factors associated with dementia risk include those associated with age-related cognitive declines, along with those associated with levels of cognitive performance, such as education. Education is also likely to play an indirect role in several of the major domains of determinants of dementia that have emerged: genetic susceptibility, vascular burden, and psychosocial and behavioral determinants that operate over the life course (Fratiglioni & Qiu, 2014). Whereas rare variants of large effect in a few genes such as amyloid β protein precursor (*APP*), presenilin-1 (*PSEN1*), and presenilin-2 (*PSEN2*) have been implicated in no more than 5% of all cases of Alzheimer’s disease, mostly early-onset alzheimer’s disease (Ballard et al., 2011), the aggregate effects of more common genetic variants with individually small effects are relevant for risk for more common, sporadic (nonfamilial) forms of the disease (Marioni et al., 2018). Estimates from twin studies put the heritability of Alzheimer’s disease at around 60% (Gatz et al., 2006). The apolipoprotein E gene (*APOE*) ε4 allele is the strongest genetic risk factor for dementia and Alzheimer’s disease identified to date (Bettens, Sleegers, & Van Broeckhoven, 2013).

However, as its status as a risk factor implies, the ε4 allele is neither necessary nor sufficient for the development of Alzheimer’s disease. Indeed, the effect of the *APOE* ε4 allele on dementia seems to be strongly attenuated by vascular health and by many psychosocial factors, such as education and mental, physical, and social activity in late life and other psychosocial factors during the entire life span (Ferrari et al., 2013; H. X. Wang et al., 2012). The vascular etiology of dementia is supported by rather strong evidence showing that a broad range of vascular risk factors and related disorders are involved in brain health (Cox et al., 2019) and in the development of both dementia and heart disease (C. Qiu & Fratiglioni, 2015; Whitmer, Sidney, Selby, Johnston, & Yaffe, 2005). Further, systematic reviews and meta-analyses have confirmed the presence of age-dependent associations of dementia with cardiometabolic risk factors such as diabetes, obesity, and hypertension and have also shown the relevance of aggregated cardiovascular risk factors including smoking, hypertension, diabetes, and hypercholesterolemia at middle age or several years before the onset of dementia (C. Qiu, 2012). Risk indices predicting risk for dementia at middle age or later in life have been developed and validated, and education (as well as unhealthy lifestyles and cardiometabolic risk factors) is a critical factor in such indices (e.g., K. J. Anstey et al., 2014; Kivipelto et al., 2006).

## Origins and Moderators of the Association Between Education and Adult Cognitive-Ability Levels

Our review thus far indicates that although the associations between educational attainment and rates of aging-related cognitive changes are on average small, inconsistent, and of limited practical relevance, the associations between educational attainment and levels of cognitive abilities are present in early adulthood and persist over time. Those findings point to the importance of understanding the developmental origins of these associations. What are the social, biological, and developmental mechanisms and modifiers of the link between educational attainment and cognitive development from childhood through early adulthood?

### Educational attainment as the outcome of a broad constellation of environmental factors

There is ample evidence to indicate that an individual’s educational attainment is partly the outcome of sociocontextual factors that operate throughout development in childhood. Parental resources, social support, and scholastic opportunities are all relevant for progression through formal schooling and ultimate educational attainment. For example, parents with greater income and education could influence their child’s access to higher education by providing greater social support, financial support, access to higher-quality primary and secondary education, and otherwise difficult-to-acquire pragmatic knowledge relevant to scholastic success (e.g., Davis-Kean, 2005; Hill & Tyson, 2009). Individual differences in educational attainment are therefore likely to be associated with the broad constellations of environmental opportunities and advantages that come with higher parental socioeconomic status (Bradley & Corwyn, 2002; Strenze, 2007). In turn, education may also influence individuals’ own socioeconomic resources and environmental exposures throughout adult life, including by partly determining occupation, social status, financial resources, and, directly or indirectly, access to quality health care (Bronfenbrenner, McClelland, Wethington, Moen, & Ceci, 1996).

Existing studies of relatively homogeneous samples from a single geographic location or even country obscure the role of structural factors in determining access to education (Glymour & Manly, 2008). Until 1954, the United States had de jure racial segregation in the states where most Blacks lived, creating profound barriers to education, and de facto racial segregation remains common in the United States. Likewise, current cohorts of older Blacks in South Africa attended school under the apartheid system, which severely limited their access to and quality of schooling (Kobayashi et al., 2019). More broadly, Lleras-Muney (2002) documented that laws regulating the age at which children could receive a work permit predicted the age at which they left school, implicating financial concerns and the opportunity cost of schooling as major drivers of educational attainment. Such concerns are relevant globally, given that the opportunity cost of keeping a physically able adolescent in school, even if school has no tuition, can be substantial. In rural communities in particular, school continuation often requires substantial travel time and related costs. Social norms also influence educational access for girls in many settings.

### Educational attainment as an outcome of individual characteristics

In addition to being the direct outcome of opportunities, educational attainment is also influenced by an individual’s own actions, behaviors, and scholastic performance (and how the individual interacts with the contextual opportunities available; Tucker-Drob, Briley, & Harden, 2013). Supporting this proposition is the consistent finding that the magnitude of individual differences in scholastic performance (and therefore also, ultimately, educational attainment) relate to traits such as cognitive abilities (Mackintosh, 2011), self-efficacy (Diseth, 2011), socioemotional skills (Mavroveli & Sanchez-Ruiz, 2011), personality (Briley, Domiteaux, & Tucker-Drob, 2014; Tucker-Drob, Briley, Engelhardt, Mann, & Harden, 2016), health (De Ridder et al., 2013), well-being (Marques, Pais-Ribeiro, & Lopez, 2011), and perception of one’s school and home environment (Hill & Tyson, 2009; Marchant, Paulson, & Rothlisberg, 2001). Cognitive ability is perhaps the most important of these: Reported correlations between general cognitive ability and scholastic achievement range from .4 to .7 (Deary, Strand, Smith, & Fernandes, 2007; Johnson, Deary, & Iacono, 2009; Mackintosh, 2011; Roth et al., 2015). Moreover, the association between cognitive ability in childhood and later educational attainment is clear (Deary & Johnson, 2010; Ritchie, Bates, Der, Starr, & Deary, 2013).

A challenge in interpreting nearly all of the observational studies cited above is that it is nearly impossible to comprehensively account for the roles of home environment, family socioeconomic status, and school and neighborhood setting. Thus, efforts to isolate the effect of any single factor, such as childhood cognitive ability, on educational attainment using observational approaches are all based on debatable and untestable assumptions. Individual characteristics related to how individuals navigate their way through the educational system are themselves outcomes of both sociocontextual environments (White, 1982) and genetically influenced dispositions (Tucker-Drob et al., 2016). The effects of childhood socioeconomic contexts on educational attainment are likely to be partly mediated by effects of those contexts on the development of psychological and behavioral characteristics relevant for both scholastic performance and educational aspirations (Sewell, Haller, & Ohlendorf, 1970). Many of the traits implicated in scholastic performance and educational attainment are heritable, which helps to explain the heritability of scholastic performance (Krapohl et al., 2014) and, ultimately, educational attainment (Cesarini & Visscher, 2017). Heritability estimates typically include both main effects of genetic factors and variance explained by gene–environment interactions, to the extent that such interactions prevail in the sample.

In a meta-analysis of 15 studies drawn from multiple countries, Branigan, McCallum, and Freese (2013) estimated an average heritability of 40% for educational attainment, with substantial heterogeneity among countries and among demographic groups within countries. They found lower heritability of educational attainment for women and for individuals born earlier in the 20th century, which is consistent with the hypothesized effect of social and structural constraints on educational attainment, discussed further below. Large-scale genome-wide association studies of education have supported the substantial heritability of education, with the most recent finding that about 15% of the between-persons differences in educational attainment are attributable to unspecified common genetic variants that are reliably measured (J. J. Lee et al., 2018).

The nature of between-persons differences in educational attainment and its psychological correlates may partly be a moving target, in that the sources of such differences may vary with age, birth cohort, period, and society (Tucker-Drob et al., 2013). The marked worldwide increase in education during the 20th century, manifested not only in the length of compulsory schooling but also in the proportion of individuals attaining upper secondary and tertiary education, has affected both the mean level and the distribution of the variable across birth cohorts. Differences between societies and changes within societies over time, such as those affecting the socioeconomic equality of educational opportunities, may introduce differences in the nature of the variance in education (Branigan et al., 2013; Heath et al., 1985; Johnson, Deary, Silventoinen, Tynelius, & Rasmussen, 2010; Rimfeld et al., 2018). For example, Heath et al. (1985) reported increased heritability of education for Norwegian men born between 1940 and 1949 compared with men born before 1940, suggesting a reduced dependency on the socioeconomic environment and an increased dependency on endogenous factors. The J. J. Lee et al. (2018) polygenic score (i.e., a value calculated on the basis of variation in multiple gene loci and their relative weights, serving as a good predictor for a trait) for educational attainment accounted for less than one sixth of the variance in educational attainment among African American participants compared with European American participants, which might be attributable to either a distinct linkage-disequilibrium structure (i.e., a correlation between alleles in different regions of DNA) in African Americans or the profound influence of social inequalities faced by African Americans in accessing education. In a similar way, historical trends in gender equality have played a major role in transforming between-persons differences in education across cohorts (Becker, 2014; Branigan et al., 2013; Buchmann, DiPrete, & McDaniel, 2008; Fischbein, Lange, & Lichtenstein, 1997; Heath et al., 1985; Weber et al., 2014).

Period effects, such as changes in the importance of education for occupational careers and in the proportion of adults who return to school later in life, might also transform how education relates to privilege and ability throughout adult life (e.g., Wilson, Zozula, & Gove, 2011). Indeed, a recent meta-analysis of genome-wide association studies of educational attainment indicated an average genetic correlation between different study cohorts of .723, well below the genetic correlation of 1.0 that would be expected if the genetic bases of educational attainment were entirely consistent across populations (J. J. Lee et al., 2018). Moreover, genetic correlations decline as the difference between the mean birth year of the cohorts increases, indicating shifts in the genetic bases of educational attainment across historical time (J. J. Lee et al., 2018). Although associations between education and cognitive performance have been observed across a wide range of societal and historical variation (Hauser & Huang, 1997; Kobayashi et al., 2017; Strenze, 2007), it may well be that such associations arise from different constellations and weights of influences, and are somewhat different in magnitude, in different societal and historical contexts.

### Effects of education on cognitive development

Beyond the effects of cognitive abilities on education, the experiences acquired during schooling may of course have effects on cognitive abilities. Though there is nothing magical about the formal school setting, it will, on average, expose individuals to more cognitive stimulation and opportunities to acquire knowledge and skills than alternative activities would. The main purpose of schooling is to train specific forms of declarative and procedural knowledge—that is, crystallized cognitive abilities. However, to the extent that knowledge is relevant for more fluid abilities (e.g., memory and reasoning)—for example, because it improves cognitive strategies and test-taking skills (Ceci, 1991)—or to the extent that the cognitive stimulation associated with schooling stimulates neurobiological change, we might expect education to affect cognitive abilities beyond crystallized abilities. Education, compared with common alternative life circumstances—such as working or being unemployed—may, on average, place greater demands on abilities such as working memory, reasoning, and declarative memory (Baker et al., 2015; Blair, Gamson, Thorne, & Baker, 2005; Wenger & Lövdén, 2016). To the extent that greater demand on these abilities during development is important for their growth, we may expect effects of education on fluid processing abilities (Lövdén, Bäckman, Lindenberger, Schaefer, & Schmiedek, 2010). Furthermore, given that curricula differ in kind across levels of the education system, longer education may provide not only more exposure but also qualitatively different exposure to opportunities and demands. Broad effects of education on cognitive abilities could also appear if education acts to protect individuals from hazards of not being in school, such as harmful effects (e.g., stress) of work environment, unemployment, or lawbreaking during childhood (e.g., Lager, Seblova, Falkstedt, & Lövdén, 2017). This may explain why the association between education and cognitive function is found across settings that differ widely in their quality of education. In settings with lower-quality educational opportunities, the hazards of not being in school may be proportionately worse. With these considerations in mind, there is good reason to hypothesize that schooling has broad effects on cognitive abilities beyond crystallized abilities.

**Fig. 5.**
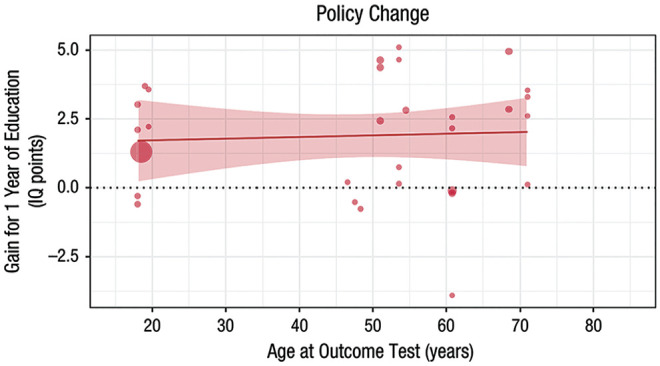
Results from a meta-analysis of the effects of 1 additional year of education on cognitive performance (IQ points), as a function of the age at which cognitive performance was measured. Effect sizes are from studies that used policy changes (e.g., increases in compulsory schooling that were implemented quasirandomly) to examine the effects of education on performance. Bubble size is proportional to the inverse variance for each estimate (larger bubbles = more precise studies). The shaded area around the regression line represents the 95% confidence interval. From “How Much Does Education Improve Intelligence? A Meta-analysis,” by S. J. Ritchie and E. M. Tucker-Drob, 2018, Psychological Science, 29, p. 1363.

In a groundbreaking review of the evidence for an effect of education on cognitive abilities, Ceci (1991) summarized evidence from several research designs, such as studies of the drop in cognitive performance during summer vacations, studies of irregular school attendance, and studies using *regression-discontinuity* methods, in which age-based cutoffs for entry into formal schooling (e.g., requirements that children be 5 years old as of September 1 to enter kindergarten) serve as a means to separate the effects of chronological maturation from the effects of 1 additional year of school. The conclusion from this review was that schooling is an important antecedent of differences in cognitive performance. More recently, Ritchie and Tucker-Drob (2018) conducted a meta-analysis of studies implementing any of three quasiexperimental methods to estimate the causal effect of schooling on cognitive abilities. Those methods included (a) regression discontinuity (e.g., Baltes & Reinert, 1969); (b) estimation of education-intelligence associations, controlling for earlier intelligence (e.g., Clouston et al., 2012); and (c) use of instrumental variables, which allowed the estimation of the effects of policies directly targeting education (i.e., increases in compulsory schooling) on intelligence (Brinch & Galloway, 2012; Lleras-Muney, 2005). All three methods produced significantly positive meta-analytic estimates. The studies of policy changes, which arguably implemented the strongest of the three methods, resulted in an average weighted effect size of about 2 IQ points (95% CI = [0.9, 3.1]), or 0.14 *SD*, per year of education (Fig. 5). That effect size is similar in magnitude to the associations between education and cognitive performance in observational studies. The mean age of the sample at the time of the postreform cognitive testing did not significantly moderate that effect, and between-study heterogeneity was observed in effect-size estimates that could not be fully accounted for by the moderators tested. This suggests that more research is needed to find factors that affect the size of education’s effect on cognitive ability.

It is noteworthy that the studies of policy changes included in Ritchie and Tucker-Drob’s (2018) meta-analysis primarily involved schooling reforms that increased the minimum number of required years of education (or, similarly, the minimum age at which students could leave school) for a given population. Thus, these studies estimated what are commonly referred to as *local average treatment effects*: the effects of increasing the duration of education for individuals who would have otherwise left school at the minimum compulsory level before the policy change. It is therefore unknown whether the effects of additional years of education after longer durations of schooling—that is, after completing college—have the same effects on cognitive performance. It is possible that as the level of education increases, schooling has diminishing marginal effects on cognitive performance—for example, (a) because learning follows an asymptotic or diminishing marginal function; (b) because people who already had higher levels of education would probably be working in cognitively complex occupations if they were not in school; or (c) because students benefit less from schooling at older ages because of declines in neurocognitive plasticity.

It is additionally instructive to consider that Ritchie and Tucker-Drob’s (2018) meta-analysis of school-age-cutoff designs produced substantially larger effect-size estimates (approximately 5 IQ points per year of education) than were produced by the meta-analysis of policy-change designs (approximately 2 IQ points per year of education). Although there are a number of potential explanations for that difference, one plausible explanation relates to differences in the timing of the outcome assessments. In studies using the school-age-cutoff design, cognitive performance was typically measured while children were still in the midst of their education. By contrast, in studies using the policy-change design, cognitive performance was typically measured several years after individuals had completed their education. Therefore, estimates from the school-age-cutoff design can be interpreted as estimates of the immediate benefits of education, whereas estimates from the policy-change design can be interpreted as estimates of the long-term benefits of education. That the school-age-cutoff estimate was more than double the size of the policy-change estimate suggests that education has effects on cognitive performance that partially fade in the initial years following school completion. Indeed, meta-analyses of early-childhood interventions indicate substantial immediate cognitive benefits that fade most precipitously in the initial years after completion of the intervention (Bailey, Duncan, Odgers, & Yu, 2017; Protzko, 2015). However, that the effect sizes for the policy-change design did not diminish with age at outcome test (see Fig. 5) may suggest that after initially fading, such effects reach a lower asymptote that is maintained into older age.

Are effects of education on cognitive development restricted to knowledge and narrow skills (e.g., reading)? Evidence suggests that this is not the case: Schooling appears to have causal effects on many different cognitive abilities. For example, in their meta-analysis, Ritchie and Tucker-Drob (2018) found significant effects of education on cognitive performance when only measures of fluid ability were considered, which indicates that education may affect cognitive performance beyond crystallized abilities, such as vocabulary, that have traditionally been associated with schooling. It is possible that education broadly affects many different cognitive abilities, including fluid abilities, by conveying narrow skills (e.g., cognitive strategies) and knowledge that are important for each of those abilities without affecting some general capacity that is relevant for all abilities. Studies addressing whether education mainly relates to narrower cognitive abilities and skills rather than to a *g* factor of cognitive abilities have generally supported this interpretation (Colom, Abad, Garcia, & Juan-Espinosa, 2002; Ritchie, Bates, & Deary, 2015; Tommasi et al., 2015). Those studies have shown that models associating education with narrower cognitive abilities fit the data better. Other evidence also supports the notion that education affects many different cognitive abilities by conveying quite narrow skills (e.g., cognitive strategies) and knowledge. For example, though we know relatively little about the effects of qualitative differences in education, some evidence suggests that the effects of different types of education (e.g., technical vs. social-science emphasis) may differ in magnitude across cognitive abilities (e.g., spatial ability vs. vocabulary; e.g., Cliffordson & Gustafsson, 2008). Those results indicate that education may operate on narrower abilities and skills rather than on general intelligence. Also relevant in this context, intellectual activities (e.g., cognitive training, music, and chess) result in limited generalization of benefits to outcome measures of cognitive performance that are not directly tapping into skills, strategies, and knowledge acquired through those activities (Melby-Lervag, Redick, & Hulme, 2016; Sala & Gobet, 2017; Simons et al., 2016).

The interpretation of this evidence depends on how the *g* factor of cognitive abilities is viewed in the first place, a matter that continues to be hotly debated (Borsboom, Mellenbergh, & van Heerden, 2003; Kovacs & Conway, 2019). If *g* is viewed as a latent trait or factor that causes differences between people in many different specific abilities, then current evidence speaks against the proposition that education is directly related to this trait. If, alternatively, *g* is viewed as an emergent property of individual differences in narrower abilities and skills that are correlated because they, for example, influence one another, then it is not a surprise that psychometric modeling fails to confirm a direct effect of education on *g.* According to the latter view, education can affect only narrow abilities, which may in turn mutually affect one another.

The mutualism model (van der Maas et al., 2006) is an example of an account of the positive correlations among different cognitive abilities (Spearman, 1904) that does not invoke a *g* factor. Whereas the *g* factor model explains that all cognitive abilities are positively correlated with one another because they are all affected by a general ability, the mutualism model instead explains these correlations as arising from mutual causation among the different abilities. K. B. Cattell (1971) hypothesized that fluid abilities are invested (e.g., via effortful cognitive processing, such as that which occurs during study) in the acquisition of crystallized knowledge. He predicted that individuals with greater fluid intelligence would acquire more crystallized knowledge (e.g., learn faster and more). Whereas Cattell’s investment model did not specifically allow for an effect of crystallized abilities on fluid intelligence, the mutualism model treats the causal process between abilities as fully bidirectional. Supporting this prediction, Ferrer and McArdle (2004) reported mutual longitudinal coupling between academic knowledge and fluid reasoning over childhood and adolescence. Ferrer, Shaywitz, Holahan, Marchione, and Shaywitz (2010) also reported mutual longitudinal coupling between reading ability and IQ in typically developing children but not in those suffering from dyslexia. Finally, Kievit et al. (2017) reported mutual coupling between vocabulary and reasoning during late adolescence. Education may play a fundamental facilitative role in these dynamic processes.

In summary, we conclude that there is strong evidence for effects of education on a broad set of cognitive abilities, including more fluid cognitive abilities. It is not yet entirely clear how to interpret these effects or gauge their relevance, but the current evidence suggests that education may affect cognitive abilities via a broad base of specific knowledge and skills that have distinct effects on many different cognitive abilities during development. In addition, it is likely that education has broad effects on cognitive abilities in part because it protects individuals from the hazards associated with not attending school.

Are the effects of education on cognitive tasks in the laboratory important and “real” in the sense that the gains are broadly applicable in everyday life and, for example, support well-being and functional independence in late life? It is possible that these effects are mediated by the effects of education on test-taking skills and do not extend to functions outside of the test situation. Though this account cannot be fully ruled out, one should note that education has robust effects on other important outcomes, including longevity (Hamad et al., 2018). Thus, though other factors correlated with education might mediate the observed positive effects on cognitive performance, prolonged education is associated with at least one outcome that all people can agree is important: life expectancy. It may be necessary for the field to move beyond the use of standard laboratory tasks as criterion measures and to incorporate alternative measures of cognitive skills that may not be captured by traditional measures but are more relevant for daily functioning and well-being. Such measures may tap specific skills needed for an individual’s idiosyncratic life circumstances (e.g., occupational skills; Ackerman, 2017) but also skills such as rationality, scientific reasoning, and decision-making (Stanovich, West, & Toplak, 2016).

### Dynamic developmental processes

We have discussed individual pathways among social contexts, educational processes, and cognitive development and how those pathways might operate in more than one direction. Such mutual effects between elements within a psychosocial system may lead to dynamic processes in which the elements reinforce one another over time. We have discussed how such dynamic processes may unfold with respect to different abilities’ effects on one another. Here, we discuss transactional models of person-environment correlation and cognitive development.

Transactional models posit that individuals differentially select, evoke, and attend to environmental experiences on the basis of differences in their abilities, interests, and motivations, and that those experiences may in turn affect those traits, thus both supporting cognitive development and increasing exposure to further environmental experiences relevant for cognitive development (Bouchard, 1997; R. B. Cattell, 1987; Hayes, 1962; Johnson, 2010; Sameroff, 1975). Because the traits that lead individuals to differentially select, evoke, and attend to environmental experiences are partly genetically influenced, these transactional processes give rise to gene–environment correlations: the tendency for individuals’ genotypes to be correlated with their environmental experiences (Beam & Turkheimer, 2013; Plomin, DeFries, & Loehlin, 1977; Scarr & McCartney, 1983).

Such active gene–environment correlations are expected to increase over the course of child development, particularly as children gain the autonomy to choose their experiences for themselves. Key to transactional models is that environmental experiences have causal effects on cognitive development, but—somewhat paradoxically—because the experiences are correlated with individual genotypes, they can cause heritability estimates for cognitive abilities to increase with age (Beam & Turkheimer, 2013; Dickens & Flynn, 2001). Indeed, there is consistent evidence of increasing heritability of cognitive abilities during development, with meta-analyses indicating increases from approximately 10% in infancy to approximately 70% by late adolescence (Briley & Tucker-Drob, 2013; Haworth et al., 2010).

Other evidence supporting transactional processes includes findings that children’s environments are “heritable” in the sense that they are correlated with their genotypes (Plomin, 1994) and reciprocal associations in prospective longitudinal studies between mental ability and parental cognitive stimulation and between children’s motivational factors and their scholastic performance (Lugo-Gil & Tamis-LeMonda, 2008; Tucker-Drob & Harden, 2012).

## What Carries the Influence of Education on Cognition Into Older Age?

To summarize the research reviewed above, educational attainment is related to levels of cognitive function throughout adulthood. However, education appears to have negligible associations with aging-related cognitive change. A model with a threshold of cognitive functioning for dementia diagnosis explains the association between educational attainment and risk for late-life dementia despite the negligible association between education and cognitive aging. Thus, educational attainment primarily influences late-life cognitive function and risk for dementia by contributing to individual differences in early adulthood that persist into old age. The developmental pathways through which education is associated with cognitive outcomes are complex and recursive. Despite that complexity, it seems safe to conclude that there is strong evidence for effects of education on both fluid and crystallized cognitive abilities. At the same time, there is also evidence for selection into longer education on the basis of cognitive ability, at least in many social contexts. Common factors that are associated with both the duration of education and cognitive ability in the course of development, such as social support, matter as well. The interplay among these educational, cognitive, and social factors during childhood and early adulthood forms individual assets and abilities that are maintained over the entire life course.

Why is it that education-related individual differences in cognitive performance that emerge in childhood and adolescence apparently fail to attenuate rates of cognitive decline in any major way? After all, greater education is associated with many favorable life conditions, including all those associated with higher socioeconomic status (e.g., increased occupational status and reduced health risk behaviors). These factors are in turn related to both cognitive performance (Hertzog et al., 2009) and dementia (Fratiglioni & Qiu, 2014), which suggests that education might have indirect effects (e.g., effects on occupational status and health risk behaviors that affect, for example, vascular status) on late-life cognitive performance and that the advantage for more highly educated individuals might increase with age. But the empirical data do not at all match such theoretical predictions. Here we note that one may also ask the opposite question: Why is it that education’s effects on cognition not fade over time, such that education-cognition associations weaken as the period of formal education recedes into the past? In fact, studies of early-childhood interventions (e.g., Head Start) before entry into formal education have demonstrated robust immediate effects on cognitive performance that fade substantially over time (Protzko, 2015).

One potential answer to either question is that the differences between people in life conditions that are established during later periods of education remain, for the majority of individuals, remarkably stable throughout the various periods of life that follow. This contrasts with the case of early-intervention programs because children enrolled in such programs often return to the same scholastic and social environment as the children in the control condition once the program has ended. In other words, the different life conditions experienced in the context of the program are not maintained over time. The situation is different in later education, especially its quality and quantity is more heterogeneous across individuals, with ample opportunity for self-selection (Sokolowski & Ansari, 2018). According to the so-called gravitational hypothesis (Gottfredson, 2003; Wilk & Sackett, 1996), people may, through self-selection and enabled access, gravitate more and more toward environments that match their cognitive abilities (Beam & Turkheimer, 2013; Tucker-Drob & Briley, 2014). The years in high school and university may be particularly relevant in this regard. After these forking paths in early adult development, individuals might, to a large extent, have found their environmental niche, such that individual differences in, for example, work conditions are considerably aligned with individual differences in cognitive abilities (to which education has contributed). The lack of more substantial effects of educational attainment on cognitive change (either positive or negative) during adulthood and older age is consistent with this view. Individual differences in cognition might reflect person-specific matches between environmental demands and abilities (see also Lövdén et al., 2010), and these matches may help to slow the fade-out of educational attainment’s effects, which are maintained in old age.

In line with these considerations, substantial evidence indicates that long-term life conditions maintain education-related differences in cognition. For example, Dekhtyar et al. (2015) collected data on school grades, occupational complexity, and dementia diagnosis in over 7,000 individuals. Higher grades were associated with reduced risk of dementia, but more so if they were followed by high occupational complexity in adulthood. As expected, a longer duration of education was protective against dementia, but controlling for occupational complexity eliminated that effect. These findings suggest that education must be leveraged into complex occupations (and convey some of its effects indirectly, through life conditions in adulthood) to protect against dementia. In fact, individuals who did not leverage their greater education into jobs that allowed for continued stimulation were not better off with respect to dementia risk than individuals with less education but more complex jobs (but see Karp et al., 2004).

Likewise, the results from a recent study using variance in the duration of education to study the effects of education on dementia risk also suggest that retaining the effects of education across the life span requires actively maintaining those effects in adulthood (Seblova, Fischer, et al., 2019). In that study, variance in the duration of education was induced by a compulsory schooling reform that extended primary school from 6 to 7 years. The reform affected 1.3 million people, resulting in high statistical power to detect effects of education on dementia risk. At the same time, it had only minor effects on students’ continued education or their socioeconomic conditions in adulthood. Presumably for that reason, the reform did not reliably affect dementia risk in old age. This highlights one of the challenges in interpreting estimates of the effects of education that is based on policy-change designs: Education may have different effects on an individual basis than it does when schooling increases for everyone in a community at the same time. If education confers benefits in part by placing an individual on a higher rung of the social hierarchy in later adulthood, the extra schooling induced by a broadly enforced legal mandate would provide little relative benefit. On the other hand, the quality of schooling of previously advantaged children might be compromised if extra resources are not provided for the larger number of students pursuing advanced education.

Social context may prevent some individuals from realizing the benefits of additional education. A handful of studies have identified settings in which increased education appears to have small or even harmful effects on certain domains of health (Courtin, Nafilyan, Avendano, et al., 2019; Courtin, Nafilyan, Glymour, et al., 2019). Current theory suggests these are settings in which structural barriers prevent individuals from translating the additional education into other resources, such as occupational success. For example, there is evidence that increases in education in the 1960s and 1970s benefited Black women in the United States more than Black men. This discrepancy is often attributed to the particularly extreme racial discrimination encountered by Black men, who found themselves blocked from many occupational paths despite their educational qualifications (Kaplan, Ranjit, & Burgard, 2008). Such barriers are unlikely to fully eliminate the association between education and cognitive skills, but they indicate that educational access alone cannot entirely offset the consequences of entrenched inequalities.

## Implications for Theories of Cognitive Aging

According to the research reviewed here, education promotes cognitive functioning and lowers dementia risk in old age, but not because it simply attenuates cognitive decline. Instead, educational attainment is associated with advantages in cognitive functioning in early adulthood that are at least partly preserved into old age. That pattern is theoretically important, not because it undermines the utility of concepts in current theories of cognitive aging or but because it constrains them.

Cognitive-reserve theory is perhaps the most influential of those theories and inspired many of the empirical studies reviewed in this article. Although there is currently no universally accepted definition of cognitive reserve (Cabeza et al., 2019; Stern et al., 2019), the concept centers around differences in how individuals’ processing of cognitive tasks shapes their susceptibility to the adverse effects of brain changes on cognitive function (Barulli & Stern, 2013; Stern, 2002, 2009, 2012). Under most definitions, the concept of cognitive reserve refers to flexible aspects of individuals’ cognitive processes that modulate how much cognitive function is influenced by brain aging, neurological disease, or injury (Stern et al., 2018). The concept as originally defined therefore also incorporates changes in cognitive processing—which some have described as compensatory (Bäckman & Dixon, 1992; Cabeza, 2002; Cabeza et al., 2018)—in response to harmful brain changes resulting from aging or disease, in addition to individual differences in processing before such changes occurred (which are sometimes referred to as *brain reserve, neural reserve*, or just *reserve* rather than compensation or cognitive reserve; Barulli & Stern, 2013; Cabeza et al., 2018; Stern et al., 2018).

Beyond referring to this concept, the term *cognitive reserve* is also commonly used to describe the theory that individual differences (a) result from education (among other factors) and (b) explain differences in cognitive function in aging. Although there is currently no measure of cognitive reserve with widely accepted construct validity (Jones et al., 2011; Nilsson & Lövdén, 2018), the proposal that education may increase cognitive reserve seems to be based on the notion that education may transmit knowledge and cognitive skills (e.g., strategies) that allow individuals to process tasks in ways that render them less susceptible to the deleterious effects of brain aging and related pathology. The brain correlates of such effects might be found in synaptic alterations rather than in macrostructural changes that would be easily detectable with current MRI techniques. In a broad sense, the cognitive-reserve concept thus implies the existence of some crystallized abilities (i.e., knowledge and skills) that can soften the impact of aging-related brain changes on cognitive functioning.

However, as stated above, although education certainly builds knowledge and cognitive skills, the available evidence does not support the claim that education, or even cognitive ability per se, is strongly associated with reductions in cognitive decline in later adulthood and older age. Such an association might arise under special circumstances. Such circumstances, however, remain to be identified in systematic ways that allow for testable predictions.

We conclude here that the current evidence is most consistent with a threshold model of the effects of education on late-life functional cognitive impairments. A person with more education will, on average, perform better in old age than a person with less. If both individuals are equally affected by aging-related brain changes or by dementia-related pathology, starting at the same age, then it will take the individual with more education longer to reach a lower threshold of cognitive functioning at which he or she is considered functionally impaired and receives a dementia diagnosis (see Fig. 4). Moreover, individuals with more education who show the same level of late-life cognitive function as individuals with less education will, on average, have experienced more aging-related declines leading up to that point. This simple threshold model suggests that individuals with more education should also show more aging-related brain changes and changes that have been strongly linked to dementia (vascular injuries, atrophy in medial temporal and parietal lobes, accumulation of tau and β-amyloid)—and should be older, on average—on diagnosis of dementia. All these scenarios have been empirically observed (see, e.g., Stern, 2006, 2009, 2012, for reviews) and can be explained without evoking the concept of cognitive reserve. Instead, they are fully compatible with the proposition that the major effect of education on late-life cognitive function is brought about through its association with cognitive-ability levels formed during childhood and adolescence and largely preserved thereafter. This conclusion is more consistent with the concept of brain reserve than with compensation or cognitive reserve (Barulli & Stern, 2013; Cabeza et al., 2018; Satz, 1993; Stern et al., 2018).

Our conclusion that education is minimally related to the rate of cognitive aging does not discredit the ambition to identify mechanisms underlying person-to-person differences in rates of cognitive aging or factors that could postpone or slow cognitive and neuronal decline. The notion of brain maintenance implies that between-persons differences in cognitive changes can be linked to between-persons differences in task-relevant aspects of the brain’s chemistry, structure, and function (Nyberg & Lindenberger, in press; Nyberg et al., 2012). That is, the less the brain changes in aging (e.g., structurally, chemically), the less cognitive ability will decline. The association is conceptually justified and has some empirical support, especially in relation to hippocampal maintenance and episodic memory (Nyberg & Lindenberger, in press; Nyberg & Pudas, 2019). Other specific examples of the broader brain-maintenance research program on cognitive aging include the *white-matter-disconnection hypothesis* (Andrews-Hanna et al., 2007; O’Sullivan et al., 2001), the *dopamine hypothesis* (Bäckman, Nyberg, Lindenberger, Li, & Farde, 2006), and the *energy-and-free-radical hypothesis* (Raz & Daugherty, 2018). More specifically, the term *maintenance* refers to the idea that certain putative factors, such as sustained engagement in physical and leisure activities as one grows older (Kohncke et al., 2016; Kohncke et al., 2018), can reduce senescent brain changes and invigorate repair processes, thereby reducing the likelihood of cognitive decline. It is conceptually and empirically clear that education is not a major contributor to these processes, given that its associations with cognitive change are minor.

## Outlook

The life-course view of education’s influence on late-life cognition that emerges from the present review warrants recommendations for future research. We believe it would be fruitful to place added research emphasis on delineating the mechanisms of cognitive development in childhood, adolescence, and early adulthood. Our review strongly suggests that investments during these earlier periods of life might reduce the individual and societal costs associated with late-life cognitive impairments and dementia—not because individuals will show less cognitive decline when they are older but because they will be able to afford more decline before reaching a threshold below which they no longer can lead independent lives. The vast majority of research on education has focused on experiences in early life (before age 25) because formal education is concentrated in that period of the life course. However, it is possible that the type of intensive educational experience characteristic of childhood would have benefits for older adults. The likely involvement of adult life conditions in maintaining initial education-related differences in cognitive ability (e.g., indirect effects of education on vascular integrity through factors such as occupational complexity and risky health behaviors) suggests this possibility. However, childhood may be a period of unusual plasticity. We do not fully understand the limits or triggers of adult neurocognitive plasticity, and cognitive interventions focusing on older adults to date have been extremely limited (i.e., in their duration or intensity) compared with typical schooling experiences.

There is also a need to better understand the cascade of events that characterize the aging of the human brain and to widen the search for measurable factors that actually do have major associations with late-life cognitive changes and the progression of diseases such as Alzheimer’s disease. The close link between early-life cognition and dementia diagnoses implies that this work must overcome substantial methodological issues, given that the cascading effects of early-life cognition are likely to induce covariance among a host of behavioral, social, and clinical factors and dementia risk. An additional challenge is evaluating the extent to which the cognitive benefits conferred by additional education spill over to other health outcomes.

The progress of research on education and cognitive aging over the past 20 years has been marked. From a public-health perspective, education appears to be one of the best-established preventive measures for dementia. There is evidence to suggest that this effect is partly causal. Furthermore, we already know how to deliver this preventive intervention, because average educational attainment can be influenced by numerous social policies. Future research can help guide public policy efforts by targeting the remaining gaps in the evidence—for example, by identifying which aspects of education matter most, pinpointing the exact direct and indirect pathways that link education to late-life cognitive functioning, delineating developmental mechanisms that reveal the degree to which timing of education is essential, and learning what accounts for heterogeneity in the effects of education on cognition in late life.

## Transparency

*Action Editor:* Nora Newcombe

*Editor:* Nora Newcombe

## Notes

1. The estimates reported here are the estimates reported by Seblova, Fischer, et al. (2019) from the analyses in which outliers were removed.

2. On December 5, 2018, we searched PubMed and Web of Science for papers (education AND dementia AND cognit* AND longitudinal) and searched through the reference lists of selected papers as well as major reviews in the field (e.g., Barulli & Stern, 2013; Karr, Graham, Hofer, & Muniz-Terrera, 2018; Meng & D’Arcy, 2012). To be included in the review of research on the progression of cognitive changes leading up to a dementia diagnosis, studies had to (a) include incidence cases in longitudinal cohort studies with careful diagnosis of dementia, (b) have some measure of cognitive performance repeatedly assessed at least 3 years before dementia diagnosis, (c) report clear procedures for dementia diagnoses that were conducted within the study (i.e., not through health records only), and (d) report analyses relevant for the question of whether education shapes the slope of cognitive decline before dementia diagnosis. To be included in the review of research on the progression of cognitive decline after dementia diagnosis, studies had to (a) include incidence cases in longitudinal cohort studies with careful diagnosis of dementia, (b) repeatedly assess cognitive performance after dementia diagnosis, and (c) report whether education was associated with the slope of cognitive decline.
